# Synthesis and Characterization of Poly(ethylene furanoate)/Poly(ε-caprolactone) Block Copolymers

**DOI:** 10.1021/jasms.4c00397

**Published:** 2025-01-09

**Authors:** Johan Stanley, Lidia Molina-Millán, Chrys Wesdemiotis, Ron M. A. Heeren, Alexandra Zamboulis, Lidija Fras Zemljič, Dimitra A. Lambropoulou, Dimitrios N. Bikiaris

**Affiliations:** † Laboratory of Chemistry and Technology of Polymers and Colors, Department of Chemistry, Aristotle University of Thessaloniki, GR-54124 Thessaloniki, Greece; ‡ The Maastricht MultiModal Molecular Imaging Institute (M4i), Division of Imaging Mass Spectrometry, Maastricht University, Universiteitssingel 50, 6229 ER Maastricht, The Netherlands; § School of Polymer Science and Polymer Engineering and Department of Chemistry, The University of Akron, Akron, Ohio 44325, United States; ∥ Faculty of Mechanical Engineering, University of Maribor, SI-2000 Maribor, Slovenia; ⊥ Laboratory of Environmental Pollution Control, Department of Chemistry, Aristotle University of Thessaloniki, GR−541 24 Thessaloniki, Greece; # Center for Interdisciplinary Research and Innovation (CIRI-AUTH), Balkan Center, GR-570 01 Thessaloniki, Greece

**Keywords:** sustainable polymers, poly(ethylene furanoate), poly(ε-caprolactone), block copolymers, MALDI-MS

## Abstract

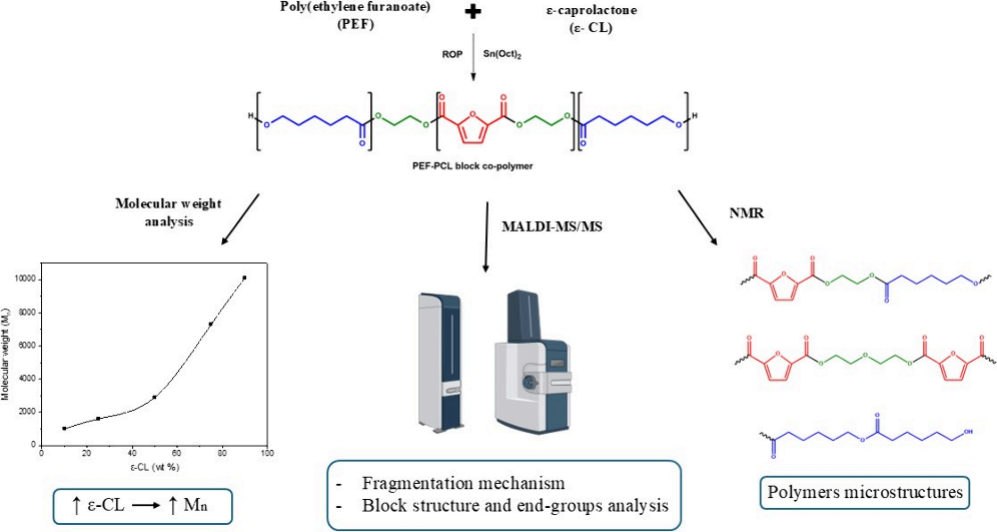

Biobased poly(ethylene furanoate) (PEF)/poly(ε-caprolactone) (PCL) block copolymers have been synthesized using ring opening polymerization (ROP) of ε-caprolactone (ε-CL) in the presence of PEF in different mass ratios. An increase in intrinsic viscosity is observed for the block copolymers with higher ε-CL content due to the extension of their macromolecular chain. Matrix-assisted laser desorption ionization time-of-flight mass spectrometry (MS) was employed to understand the composition and structure of the produced block copolymers. The MS analysis helped to confirm the formation of PEF–PCL copolymers in all cases. Furthermore, tandem mass spectrometry experiments were performed to analyze the intrinsic fragmentation mechanism of neat PEF and PCL (both linear and cyclic) and confirm the block structure and end-groups. Finally, nuclear magnetic resonance results confirmed the composition and microstructure of the block copolymers. The synthesized PEF–PCL block copolymers can be used as a replacement for petroleum derived plastics, especially in the field of food packaging.

## 1 Introduction

In polymers, like in most materials, properties are a direct consequence of the chemical structure and molecular weight. Therefore, several strategies have been developed to control those as well as various characterization techniques in order to determine them. Especially in copolymers, it is important to determine their microstructure, i.e., block or random, as this further affects their properties. In comparison to random copolymers, block copolymers typically have better physical and mechanical characteristics. The ability to modify a crystalline polymer without significantly lowering its melting point, modulus, tensile strength, or elongation, along with the ability to “build in” a specific property through the appropriate choice of a second component, are some advantages of block copolymers over random copolymers.^1^ Popular techniques to determine the microstructure and average molecular weight include end-group analysis by titrimetry, measurement of intrinsic viscosity, nuclear magnetic resonance (NMR), and size exclusion chromatography (SEC). A relatively new method in the study of polymer structure is matrix-assisted laser desorption ionization time-of-flight mass spectrometry (MALDI-TOF-MS).

Indeed, MALDI-TOF-MS, an analytical technique that is used for the characterization of complex mixtures, has proven very powerful in the field of polymer science.^2^ MALDI-TOF MS facilitates the identification of chain composition and end-groups with simple sample preparation. It is also employed to accurately determine molecular weight distributions of polymers with low polydispersity indices (PDI < 1.2).^3^ Unlike other common techniques for polymer characterization, such as SEC and viscometry, MALDI-TOF-MS offers information not only about average molecular weight (*M*_n_ and *M*_w_) but also about the exact mass of repeating units and end-group masses. Moreover, MALDI-TOF-MS overcomes obstacles posed by other typical end-group analysis techniques, including nuclear magnetic resonance (NMR) and infrared (IR), when analyzing polymers with *M*_w_ higher than a few kDa.^4^ In NMR, only a small fraction of the signal corresponds to the end-groups, which is especially noticeable for high molecular weight polymers. By contrast, in MALDI-TOF-MS, all detected signals contain end-group information, resulting in a significantly enhanced overall end-group signal compared with NMR. Hence, MALDI-TOF-MS is particularly suitable for understanding end-group functionalities and gaining further insights into synthesis mechanisms. The combination of MALDI-MS with tandem MS further enhances the structure analysis of homopolymers and copolymers.^5^

In MALDI, the matrix cocrystallizes with the polymeric sample. Then, the matrix absorbs the laser energy and transfers it to the analyte molecules, causing them to desorb and ionize. The analyte molecules are subsequently separated based on their mass-to-charge ratio (*m*/*z*) and detected by the mass analyzer. Although using MALDI-TOF-MS for analyzing copolymers can be challenging due to spectral complexity and potential isobaric interferences,^2^ multiple studies have proven this technique is invaluable for the structure and sequence characterization of copolymers.^6−8^ Furthermore, other studies have highlighted the benefits of combining MALDI-TOF-MS with SEC and NMR analysis for a more comprehensive characterization of copolymers.^5,9−11^ In the present study, MALDI-TOF-MS analysis complemented by tandem MS spectrometry will be applied to investigate the structure of poly(ethylene furanoate)/poly(ε-caprolactone) copolymers that were synthesized as environmentally friendly copolymers for potential food packaging applications.

Over the last few decades, concern over environmental issues is increasing. This includes the effects of rising greenhouse gas (GHG) emissions from fossil fuel combustion. Since fossil fuels are the primary energy source in conventional plastics manufacturing, their production and use contribute to this side of the problem. Therefore, it is essential to identify sustainable substitutes for conventional fossil-based polymers.^12^ Biobased polymers are greener than fossil-based ones as fossil carbon in the production process is replaced by renewable carbon from biomass, which is essential for both eco-friendliness and sustainability.^13^ Among biobased polymers, some are nonbiodegradable, such as biobased poly(ethylene terephthalate) (PET), poly(ethylene furanoate) (PEF),^14^ and biobased polyolefins, and some, such as poly(lactic acid) (PLA),^15^ poly(ε-caprolactone) (PCL), and polyhydroxyalkanoates (PHAs), are biodegradable.^16^

**Figure 1:**
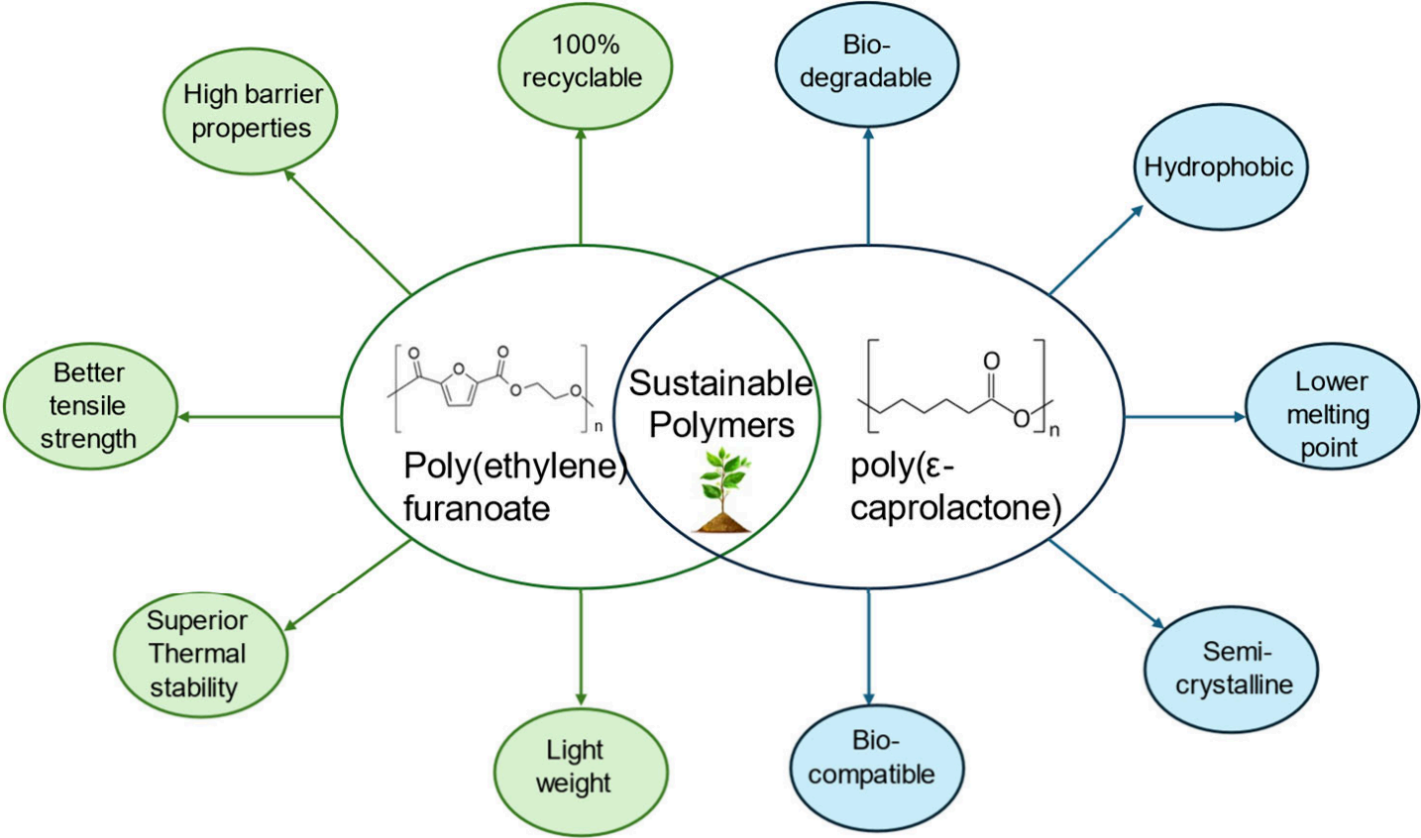
Properties of PEF and PCL.

PEF is a 100% biobased polymer that has received interest from industry and academia owing to its superior thermal properties (higher glass transition temperature (*T*_g_) (85–95 °C) and lower melting point (*T*_m_) (210–215 °C)) and improved barrier properties (higher oxygen, carbon dioxide, and water barrier) in comparison to PET, as shown in Figure 1.^17^ PEF is synthesized using 2,5-furan dicarboxylic acid (FDCA) or its derivative dimethyl 2,5-furan dicarboxylate (DMFD) and ethylene glycol (EG). Both of these monomers can be produced from lignocellulosic biomass.^18^ When compared to PET, the manufacturing of PEF can lower nonrenewable energy use (NREU) by 40% to 50% and greenhouse gas (GHG) emissions by 45% to 55%.^19^ Similar to the PET recycling process, PEF can also be recycled both mechanically and chemically. Moreover, PEF can be separated from other types of plastics using near-infrared (NIR) technique, which is a precondition for PEF recycling cycle.^20^

Although PEF is recognized for its excellent mechanical strength, high stiffness, and great barrier properties, its low ductility limits its use in applications requiring flexibility such as stretchable films or flexible packaging. Additionally, while biobased, PEF is not biodegradable under natural conditions. To address these limitations, copolymerizing PEF with ductile and biodegradable materials such as PCL has shown promising results. For example, studies on poly(butylene 2,5-furandicarboxylate)-*block*-poly(caprolactone) copolyesters highlight how PEF provides strength and thermal stability, while PCL enhances flexibility and biodegradability. It has been reported that increased PCL content improves chain dynamics, elongation at break, and flexibility, while reducing hardness and tensile strength. Modified PEF (such as copolymers, blends, and composites) offers excellent mechanical properties, and thermal resistance and dimensional integrity makes it suitable for a wide range of industrial applications as a replacement for fossil-based consumer goods, such as packaging materials, fibers for textiles, automobile sectors, and electrical and electronic applications.^21−24^

PCL is a linear aliphatic polyester extensively studied for tissue engineering, drug delivery, and sustainable packaging applications. It is mainly synthesized by ionic and metal catalyzed ring opening polymerization (ROP) of ε-caprolactone (ε-CL).^25,22^ It has a low melting temperature (*T*_m_) (∼60 °C) and a very low glass transition temperature (*T*_g_) (about −60 °C). Importantly, despite a slow degradation rate, PCL is a biodegradable polymer, since the polymer backbone is hydrolyzable at the ester linkages.^26^ Aromatic–aliphatic copolymers with PCL have been synthesized to impart a higher flexibility to the aromatic counterpart while maintaining high tensile strength. A second point of interest is the possible increase in the biodegradability of the aromatic polymer.

However, to the best of our knowledge, there has been only one published work on PEF/PCL copolymers. In that study, random copolymers were obtained by reacting PEF oligomers with ε-CL (20 to 70 mol %), in a three-stage procedure. PEF oligomers were initially prepared by the esterification of FDCA with ethylene glycol (EG), then ε-CL ring opening polymerization was carried out, and in the end, a polycondensation step was performed under vacuum. The resulting copolyesters showed random microstructures, amorphous nature, one *T*_g_, and lower thermal stability than neat PEF. Interestingly, the introduction of ε-CL moieties improved the chain mobility and imparted copolyesters with enhanced mechanical performance.^27^

As could be expected, MALDI-TOF-MS analysis has also been used to investigate furan-based polymers and copolymers,^28−31^ in most cases aiming to gain insight on the synthesis of oligomers or low molecular weight polymers. In these studies, end-group analysis contributed to determining whether linear or cyclic adducts were obtained, their size, and whether the polymer chains were terminated by OH, COOH, COOR, or other groups, giving overall valuable insights on the structure of the studied polymers. Saar et al.^32^ used MALDI-TOF-MS analysis to confirm postpolymerization functionalization, while Maaskant et al.^33^ employed it to study the UV degradation of PEF. Papodopoulos et al.^34^ for the first time used liquid chromatography (LC) coupled with high resolution mass spectrometry (HRMS) to understand the structure of PEF oligomers (formation of dimers and trimers) during the first step of the polymerization process.

The novelty of this study lies in its innovative approach to synthesizing PEF–PCL block copolymers through ROP of ε-caprolactone in the presence of PEF, exploring various mass ratios to potentially enhance polymer properties. The research employs advanced characterization techniques, including MALDI-TOF-MS and MALDI-MS/MS, to confirm the formation and analyze the structure of the block copolymers, providing insights into their composition, block structure, and end-groups. Additionally, NMR is used to verify the composition and microstructure, highlighting the importance of block copolymers for improved physical and mechanical properties. The study proposes these synthesized copolymers as sustainable alternatives to petroleum-derived plastics, broadening their industrial application potential, particularly in food packaging.

## 2 Materials and Methods

### 2.1 Reagents and Solvents

2,5-Furandicarboxylic acid (BioFDCA X000230-2003) was purchased from Corbion (Gorinchem, The Netherlands). Ethylene glycol (anhydrous, 99.8%) and antimony trioxide (Sb_2_O_3_) were purchased from Aldrich Co. (London, UK); ε-caprolactone (ε-CL, purity 99%) and stannous octoate (Sn(Oct)_2_) used as catalysts were purchased from Sigma-Aldrich (Saint Louis, MO, USA). LC-MS grade methanol (MeOH) was acquired from Biosolve Chemie B.V. (Valkenswaard, The Netherlands). Hexafluoroisopropanol (HFIP, ≥99% purity), 2,5-dihydroxybenzoic acid (DHB), and sodium chloride (NaCl, ≥99.5% purity) were supplied by Sigma-Aldrich Chemie B.V. (Zwijndrecht, The Netherlands). All organic solvents were used without further purification.

### 2.2 Synthesis of PEF–PCL Block Copolymers

PEF was synthesized by using a two-stage melt polycondensation method. FDCA and EG were utilized at a 1:2.1 molar ratio in the first step (esterification). The reaction flask was evacuated and filled three times with nitrogen to eliminate air. The reaction mixture was then preheated under nitrogen flow for 30 min at 170 °C and for an hour at 190–200 °C at a stirring speed of 200 rpm. H_2_O distillation usually occurs within 1–1.5 h of the first step. Before the second step (condensation), Sb_2_O_3_ catalyst (300 ppm) was introduced into the reaction flask, and vacuum (5.0 Pa) was applied slowly for 15 min. The reaction was further maintained under vacuum for 6 h while the temperature was progressively raised to 250–260 °C. The stirring speed was reduced (100 to 70 to 50 rpm) to avoid high shear stress, and finally the samples were retrieved and characterized.

**Scheme 1:**
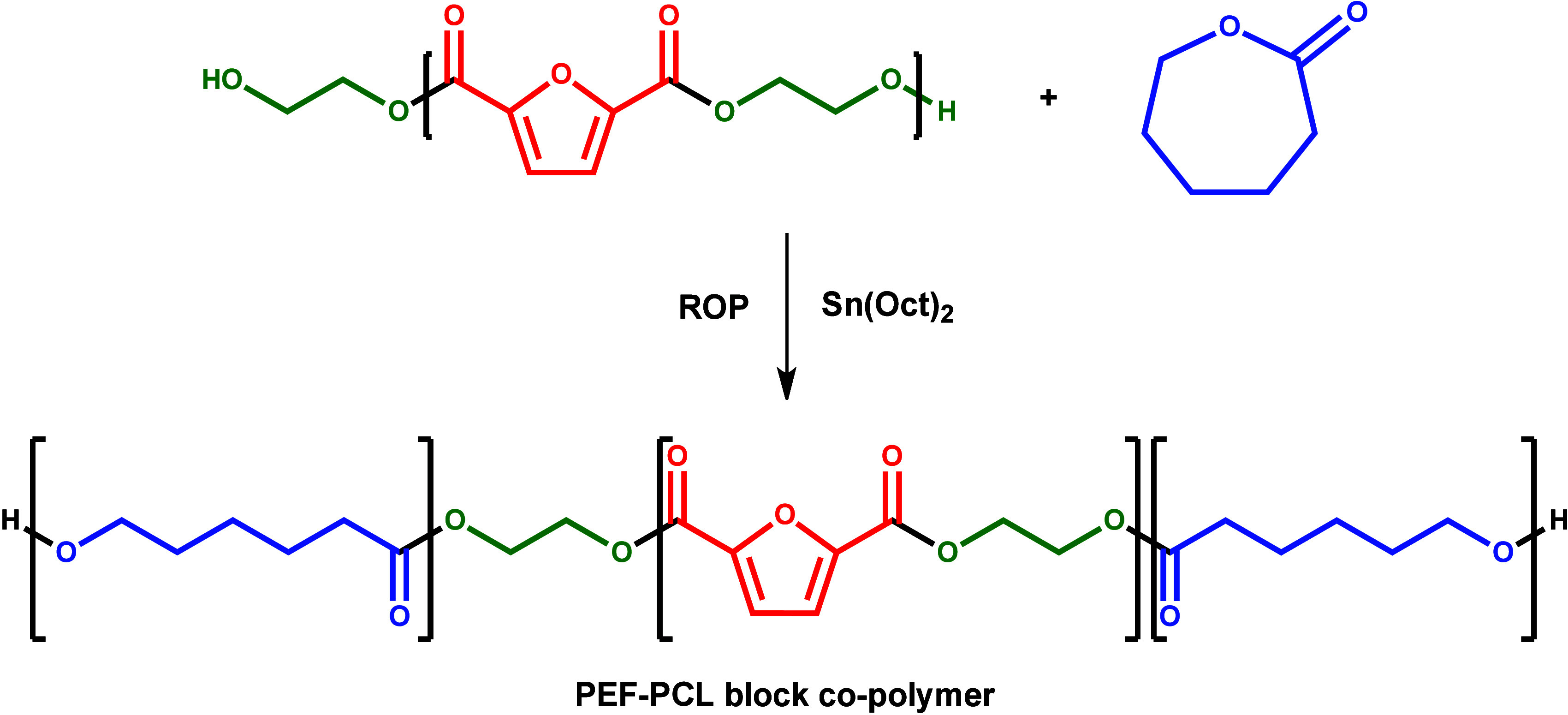
Ring Opening Polymerization (ROP) of ε-Caprolactone to Synthesize PEF–PCL Block Copolymers

PEF/PCL copolymers with various mass ratios, such as 10/90, 25/75, 50/50, 75/25, and 90/10 w/w, were synthesized using a melt polycondensation technique by adding proper amounts of PEF and ε-CL monomer as displayed in Table 1. The copolymers were synthesized at different mass ratios to explore how varying the composition affects the structural properties of the resulting block copolymers. By adjusting the ratios, we aimed to investigate the impact on intrinsic viscosity and molecular weight and optimize its properties for food packaging applications. Ring opening polymerization of ε-CL took place at 200 °C under a nitrogen flow and a stirring rate of 300 rpm. Sn(Oct)_2_ was added as a catalyst (0.5 wt % of added materials), and the reaction was completed after 2.5 h. The synthesized copolymers were retrieved and characterized. The synthesis route of PEF–PCL block copolymers is illustrated in Scheme 1.

**Table 1:** Workflow Summary of the Experimental Design Steps Followed to Synthesize and Analyze PEF–PCL Block Copolymers

step	description	outcome
1	synthesis via ROP of ε-caprolactone with different mass ratios	PEF–PCL block copolymers
2	intrinsic viscosity analysis	characterization; physical properties information
3	MALDI-MS analysis	characterization; confirmation of PEF–PCL block copolymers formation
4	MALDI-MS/MS analysis	fragmentation patterns and mechanisms; end-group and comonomer sequence characterization
5	NMR	characterization; microstructure and segment lengths of block copolymers

### 2.3 Characterization

#### 2.3.1 Intrinsic Viscosity Measurement

Intrinsic viscosity (*η*) measurements were performed using an Ubbelohde viscometer at 30 °C in a mixture of phenol/1,1,2,2-tetrachloroethane at a ratio of 60/40 w/w. Sample concentrations of 1% (w/v) were used. The [*η*] value of each sample was calculated using the Solomon–Ciuta equation:^35^
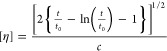
1where *c* is the concentration of the solution, *t*_0_ is the flow time of pure solvent, and *t* is the flow time of the solution. Three different measurements were made on each sample, and the average value was computed to ensure the data accuracy.

The *M*_n_ of the samples was determined using the Berkowitz equation:^18^
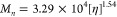
2

#### 2.3.2 MS Sample Preparation

**Figure 2:**
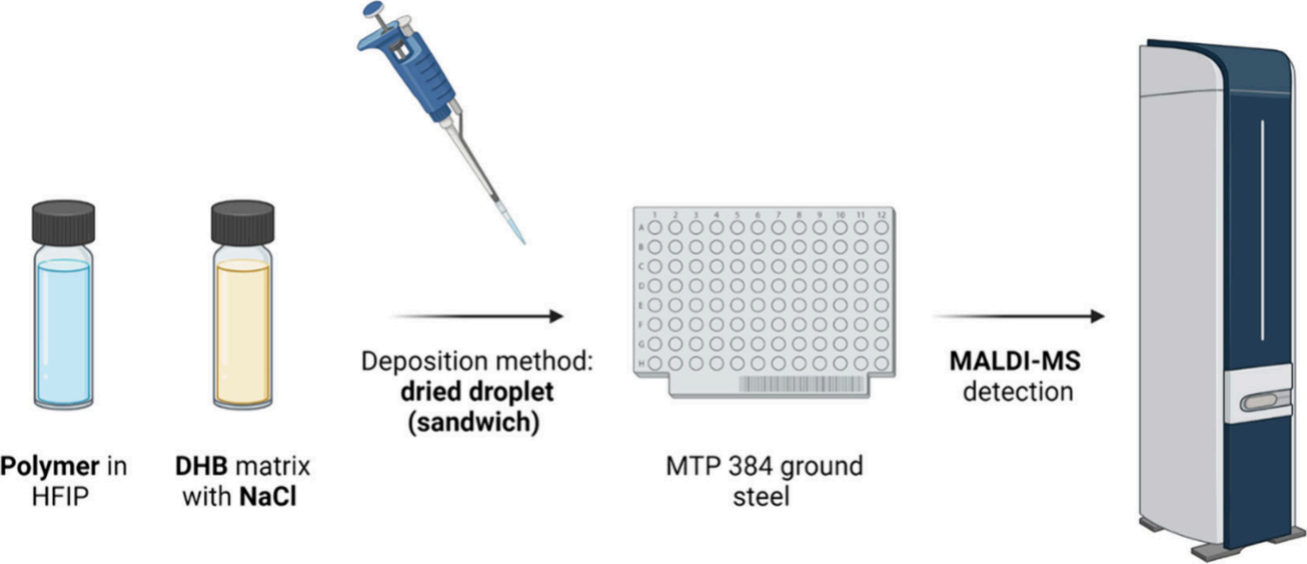
Pictorial representation of sample preparation for MALDI-MS detection.

Stock solutions of PEF, PCL, and copolymers were prepared to a final concentration of 2 mg/mL in HFIP. DHB matrix (15 mg/mL, MeOH) was mixed with NaCl (3 and 0.6 mg/mL for MALDI-MS and MALDI-MS/MS, respectively). The sandwich method was used for sample deposition on a MTP 384 ground steel target.^36,37^ Briefly, 0.5 μL of DHB/NaCl solution were deposited and air-dried on the target plate. Next, 0.5 μL of the polymer stock solutions were pipetted on top of the first DHB/NaCl layer. After evaporation, a second layer of matrix (0.5 μL of DHB/NaCl) was deposited on top of the mixture. The target plate was air-dried prior to analysis. Figure 2 displays the sample preparation steps for the MALDI-MS analysis.

#### 2.3.3 MALDI-MS

MALDI-MS measurements were performed to elucidate the structural characteristics and composition of the synthesized block copolymers. Polymer and copolymer samples were measured on a RapifleX Tissuetyper (Bruker Daltonics GmbH & Co. KG, Bremen, Germany) operating at 5000 Hz, 90% laser power, and 500 shots per pixel. Measurements were conducted in positive ion mode with a mass range of *m*/*z* 100–3000. Calibration of the *m*/*z* scale was performed with red phosphorus in positive-ion mode prior to the acquisition.^38^ FlexControl and flexImaging (Bruker Daltonics) software were used for MSI data acquisition.

#### 2.3.4 MALDI-MS/MS

MALDI-MS/MS analysis was conducted on all samples previously examined by MALDI-MS to obtain comprehensive structural information about block copolymers. This technique enabled the study of fragmentation patterns and facilitated detailed end-group and comonomer sequence analysis, providing deeper insights into the molecular architecture of the synthesized block copolymers. A timsTOF fleX (Bruker Daltonics) was used for all of the MS/MS experiments. All spectra were collected in positive-ion mode between 200–3500 *m*/*z*. Calibration of the *m*/*z* scale was performed with red phosphorus in positive-ion mode prior to the acquisition. FlexControl and flexImaging (Bruker Daltonics) software were used for data acquisition. All measurements were performed with the following settings: 1000 Hz repetition rate, 5000 laser shots per pixel, and 88% laser power. Precursor ions were isolated using a 4 *m*/*z* isolation window and fragmented with collision energies ranging from 60 to 100 eV. Certain MS/MS data were acquired as a summation of spectra to ensure signal quality. Data analysis was performed using the Compass DataAnalysis program (Bruker Daltonics).

#### 2.3.5 Nuclear Magnetic Resonance (NMR)

The block copolymers were further analyzed using NMR to confirm their composition and microstructure in support of the findings from MALDI-MS and MALDI-MS/MS. NMR provided additional details about the copolymer backbone and segment lengths, improving our understanding of the microstructure of the synthesized block copolymers. The copolymers were dissolved in deuterated chloroform (CDCl_3_)/deuterated trifluoroacetic acid (TFA-*d*_1_) mixtures. Nuclear magnetic resonance (NMR) spectra were recorded on an Agilent 500 spectrometer and calibrated using the residual CDCl_3_ solvent peaks.

The degree of randomness *B* and the average sequence length of the PEF, *L*_EF_, and PCL, *L*_CL_, segments were calculated according to the following equations,^39^ where *F*(CLCLCL) is the value of the CLCLCL triad fraction, calculated from the corresponding peaks in the ^13^C NMR spectra, *X*_EF_ and *X*_CL_ are the molar fractions of PEF and PCL, respectively, and α is a parameter depending on the composition, calculated from the first equation.
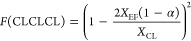
3
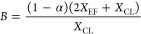
4
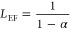
5
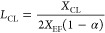
6

## 3 Results and Discussion

### 3.1 Synthesis and Characterization

A series of PEF–PCL block copolymers has been synthesized using ROP of ε-CL with different mass ratios of PEF, as displayed in Table 2. The intrinsic viscosity (*η*) values of neat PEF and neat PCL were 0.64 and 0.78 dL/g, respectively, and for the block copolymers ranged from 0.23 to 0.63 dL/g. The number average molecular weight (*M*_n_) determined using eq 2 shows values ranging from 1000 to 10,100 g/mol for the block copolymers. According to the literature, polyesters produced via melt polycondensation have a low molecular weight probably due to the simultaneous, competing reactions of condensation and degradation. Additional methods like chain extension or solid-state polycondensation are typically used to further increase the molecular weight of the polyesters.^40^

**Table 2:** Intrinsic Viscosity and Molecular Weight Determination

name	PEF/PCL ratio	[*η*] (dL/g)	*M*_n_ (g/mol)
PEF	100/0 (neat PEF)	0.64	11,400
P9010	90/10	0.23	1000
P7525	75/25	0.28	1600
P5050	50/50	0.36	2900
P2575	25/75	0.53	7300
P1090	10/90	0.63	10,100
PCL	0/100 (neat PCL)	0.78	18,250

**Figure 3:**
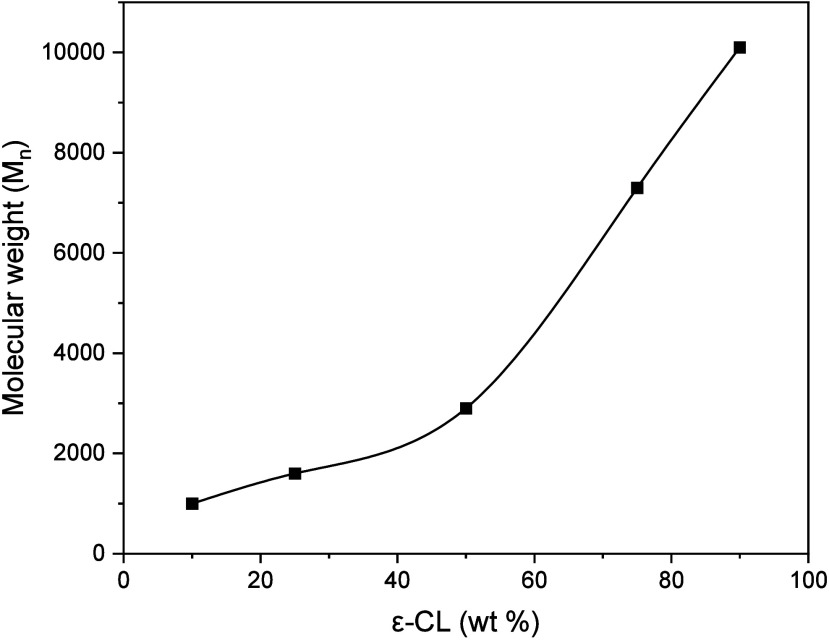
Molecular weights of synthesized block copolymers vs wt % of PEF.

From the results, it is observed that copolymer samples with higher ε-CL content possess higher intrinsic viscosity. The P1090 sample with 90% ε-CL content shows higher intrinsic viscosity among the synthesized block copolymers. The PEF and ε-CL polymerization mechanism indicates that macromolecular chains become extended with an increase in the ε-CL concentration. The length of the polymer chain affects the intrinsic viscosity of the polymer. A longer polymer chain will result in more entanglements between the chains and thus a higher intrinsic viscosity value.^41^ The block copolymers synthesized were expected to be mainly A–B–A type according to the reaction shown in Scheme 1, where A is the PCL segment generated during ring opening polymerization and B is the initial PEF present in the middle of the macromolecular chain. The proportion of macromolecular chains with hydroxyl end-groups decreases with a reduction in initial PEF concentration, at which only a few hydroxyl groups are available to function as initiators for ROP of the ε-CL monomer. Therefore, it is anticipated that part B’s dimensions will stay constant throughout all block copolymers, whereas part A’s dimensions will grow as the ε-CL content increases. The molecular weights (*M*_n_) of the block copolymers increase with ε-CL content as displayed in Figure 3. However, the presence of carboxyl groups is proof that degradation happened during the polycondensation reaction. Hence, other types of block copolymers, as well as random copolymers, are expected to less extent.^40^

### 3.2 MALDI-TOF (MS and MS/MS) Analysis

**Figure 4:**
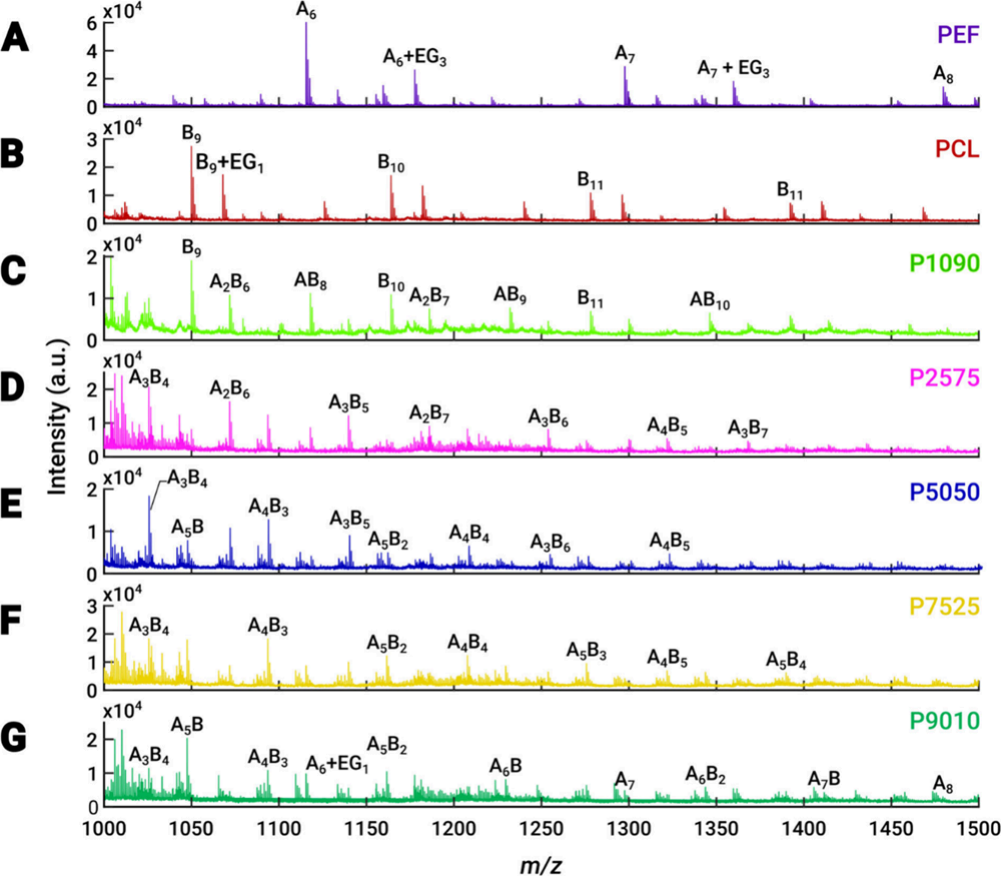
MALDI-TOF mass spectra of neat (A) PEF and (B) PCL polymers. (C–G) Mass spectra of PEF/PCL copolymers with different mass ratios. The spectra highlight cyclic and linear polymer peaks. In the spectra, A denotes PEF and B denotes PCL. EG_1_ (water, H_2_O) and EG_3_ (ethylene glycol, C_2_H_6_O_2_) correspond to the end-groups attached to the main chain.

In this work, we employed MALDI-TOF-MS to analyze neat PEF and PCL polymers and PEF–PCL copolymers in positive ion mode. Figure 4 shows the mass spectra of the polymers and copolymers within a mass range of 1000–1500. The main objective was to confirm the successful synthesis of the copolymers and to gain deeper insights into their structure and end-groups.

The spectrum of neat PEF (Figure 4A) comprises [M + Na]^+^ ions separated by 182 Da, corresponding to the PEF monomer unit. The abundance of the sodiated ions is attributed to the presence of NaCl as a cationizing agent. While the most intense peaks correspond to the cyclic polymer, some signals from the linear form were also detected at *m*/*z* 1177.6 and *m*/*z* 1359.6. The linear form of PEF corresponds to a PEF chain with H– and −CH_2_CH_2_OH end-groups (i.e., ethylene glycol, EG_3_:62 Da, see Table 3).

**Table 3:**
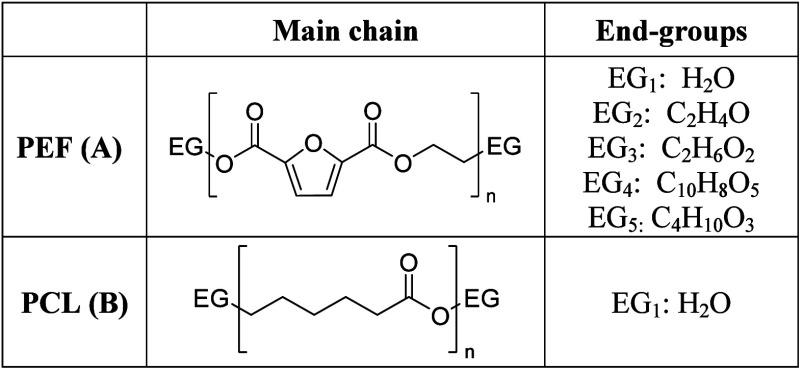
Structure of the Monomeric Units of PEF (A) and PCL (B) Polymers and Their Respective End-Groups

Similarly to PEF, the acquired MALDI spectrum for neat PCL (Figure 4B) shows a homologous series of Na adducts separated by 114 Da (PCL monomer unit mass), as expected. Again, the most intense ions arise from the cyclic polymer, but linear PCL (H– and −OH end-groups) was also detected (peak at *m*/*z* 1068.0, see Table 3). A list of theoretical *m*/*z* values and identities for the main ions formed from PEF and PCL are provided in Tables S1 and S2.

These results indicate that both neat polymers consist of a mixture of linear and cyclic forms. Although the viscosity data indicate that the neat PEF and PCL polymers prepared in this study have high molecular weight, the ions detected with MALDI correspond to short-chain oligomers. This could be due to the underestimation of the molecular mass average by the MALDI technique, which is more efficient for the low mass oligomers.^42^ Indeed, low *M*_w_ chains are better ionized and more highly detected than the higher *M*_w_ chains.

For the block copolymer spectra, different trends are observed depending on the mass ratio of PEF/PCL employed during the synthesis. For instance, the spectra of block copolymers of high content of PCL (Figure 4C) display high intensity signals of copolymers with numerous monomeric units of PCL. Similarly, the spectra of block copolymers of high content of PEF (Figure 4G) show the abundance of synthesized blocks with multiple monomeric units of PEF. In these spectra, we can also observe oligomers composed of only the neat polymers, such as *m*/*z* 1049 corresponding to a PCL chain of nine monomers in Figure 4C or *m*/*z* 1133.5 corresponding to a PEF of six units in Figure 4G. This suggests that the synthesis products contain a mixture of both block copolymers and homopolymers, derived from the starting material. For the block copolymers with other mass ratios (Figures 4D–4F), the spectra mainly show signals of the formed block copolymers. According to our results in Figure 4, the block copolymers formed are not very long chains, which aligns with the intrinsic viscosity and the *M*_n_ values for these compounds.

Overall, we have confirmed the synthesis of PEF and PCL block copolymers with different mass ratios using MALDI. MALDI-TOF-MS/MS was employed to provide further insights into the structure and end-groups of the formed block copolymers. However, this technique may not provide detailed insights into block size or arrangement. Additional analysis was performed using NMR to understand the segment lengths and microstructures of the block copolymers.

**Figure 5:**
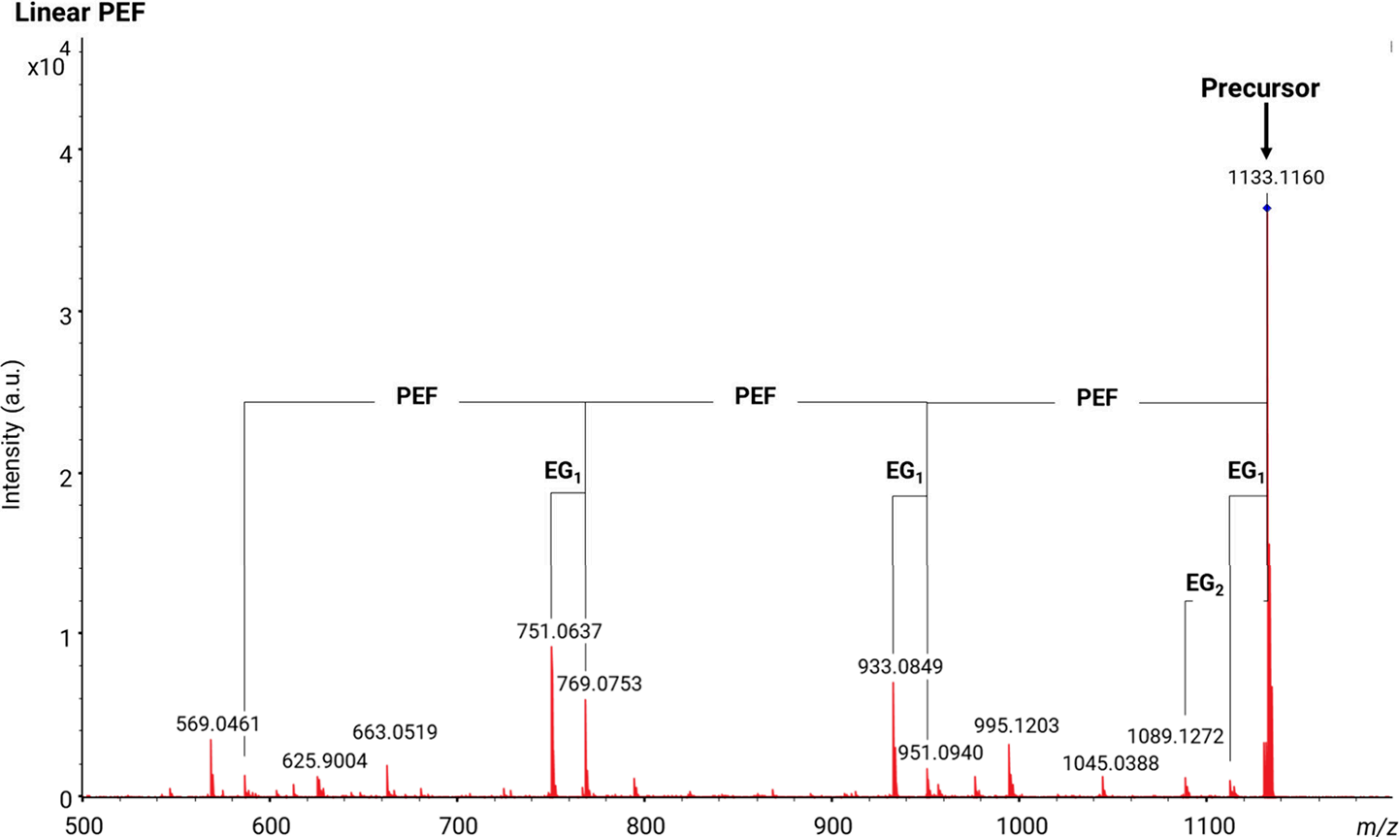
MALDI-MS/MS spectrum of the [M + Na]^+^ ions from linear PEF polyester (*m*/*z* 1133) showing fragment series from loss of EG_1_ or EG_2_ and *n* repeat units (see Scheme 2).

Tandem mass spectrometry was employed to determine the block length and copolymer composition since it offers insight into the microstructure of polymer chains. In our present work, mass spectrometry has been utilized to confirm the copolymer formation, and tandem approaches have demonstrated the ability to qualitatively depict the compositional characteristics of copolymers and identify their category (random/block/gradient, etc.).^43,44^Figure 5 shows the MALDI-MS/MS CID spectrum of the synthesized linear PEF polyester. The oligomer at *m*/*z* = 1133 was selected. The fragments observed lost repeating units of PEF and one of the main end-groups, EG_1_ or EG_2_. Theoretical *m*/*z* values for the monomeric units of PEF and PCL with various end-groups are provided in the supplementary section (Table S3).

**Scheme 2:**
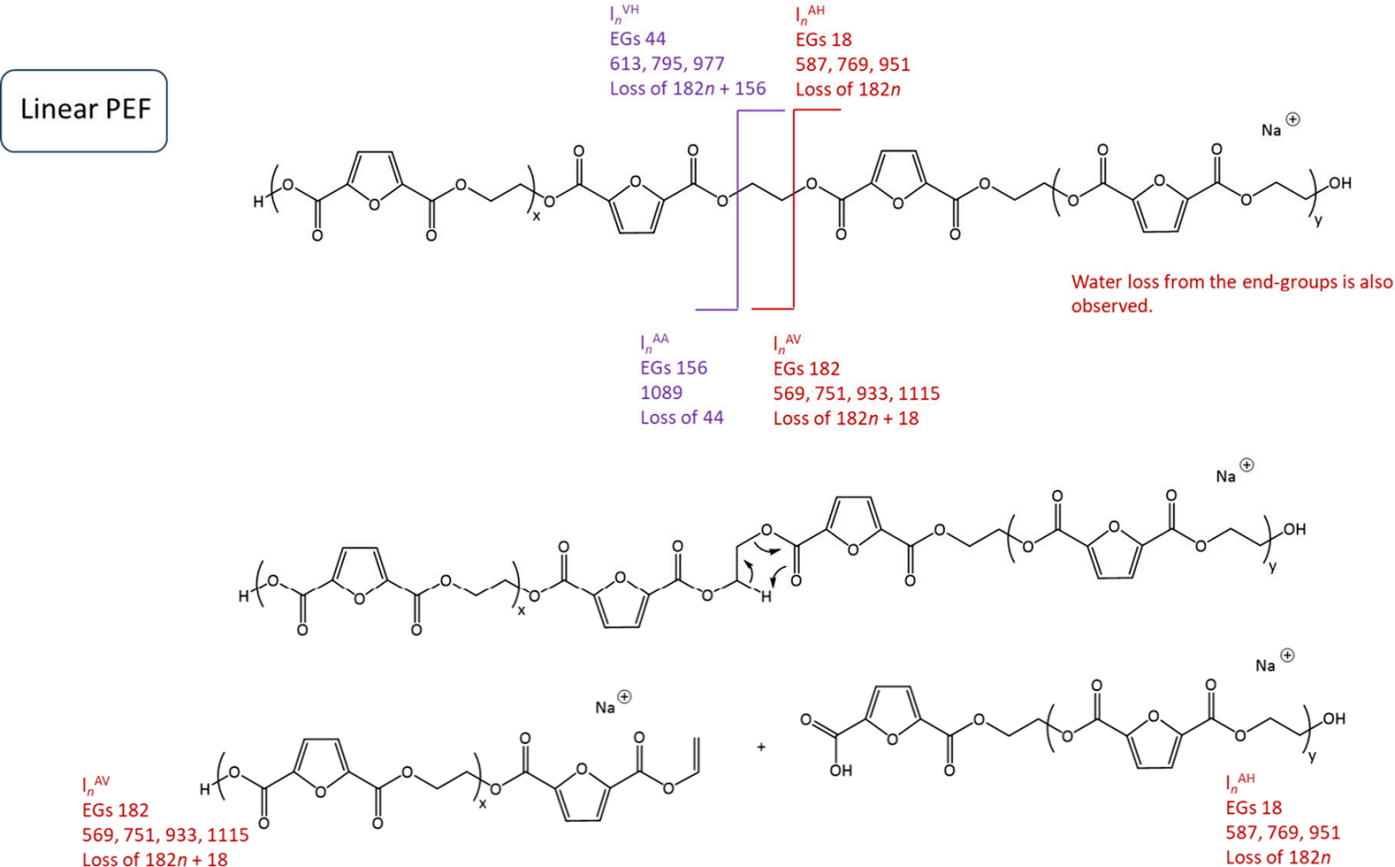
[M + Na]^+^ Linear PEF Polyester and Proposed Fragmentation Mechanism Involving 1,5-H Rearrangement at the Ester Groups and Leading to Linear Fragments End-groups given in superscripts. The *m/z* values of the main fragments are displayed in the scheme. *l*_n_, linear fragments; AV, vinyl acid; AH, acid hydroxyl; VH, vinyl hydroxyl; AA, diacid.

The fragments from the linear PEF polyester can be explained by a McLafferty rearrangement (1,5 hydrogen transfer) over the ester bond leading to COO–C bond cleavage, as illustrated in Scheme 2.^10^ The linear PEF polymeric chain has been broken into two fragments with a vinyl end-group at one end and a hydroxyl end-group at another end. It is also noted that the end-groups lose water molecules. The fragments retaining the initiating chain end contain shorter linear chains with vinylhydroxyl (VH) and diacid (AA) end-groups. Conversely, the terminating chain end is contained in shorter linear fragments with vinyl acid (AV) and acid hydroxyl (AH) end-groups.

**Figure 6:**
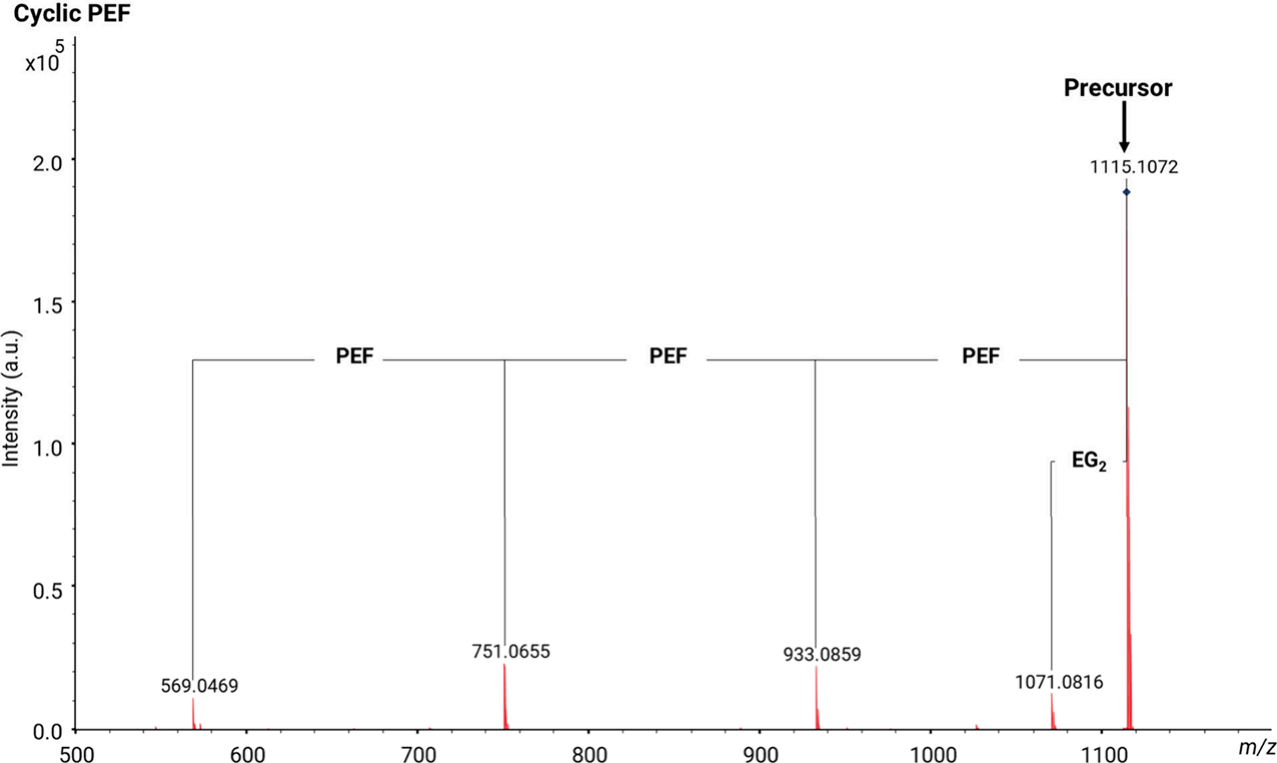
MALDI-MS/MS spectrum of [M + Na]^+^ from cyclic PEF polyester (*m*/*z* 1115) showing fragments arising by the loss of EG_2_ or *n* repeating units.

**Scheme 3:**
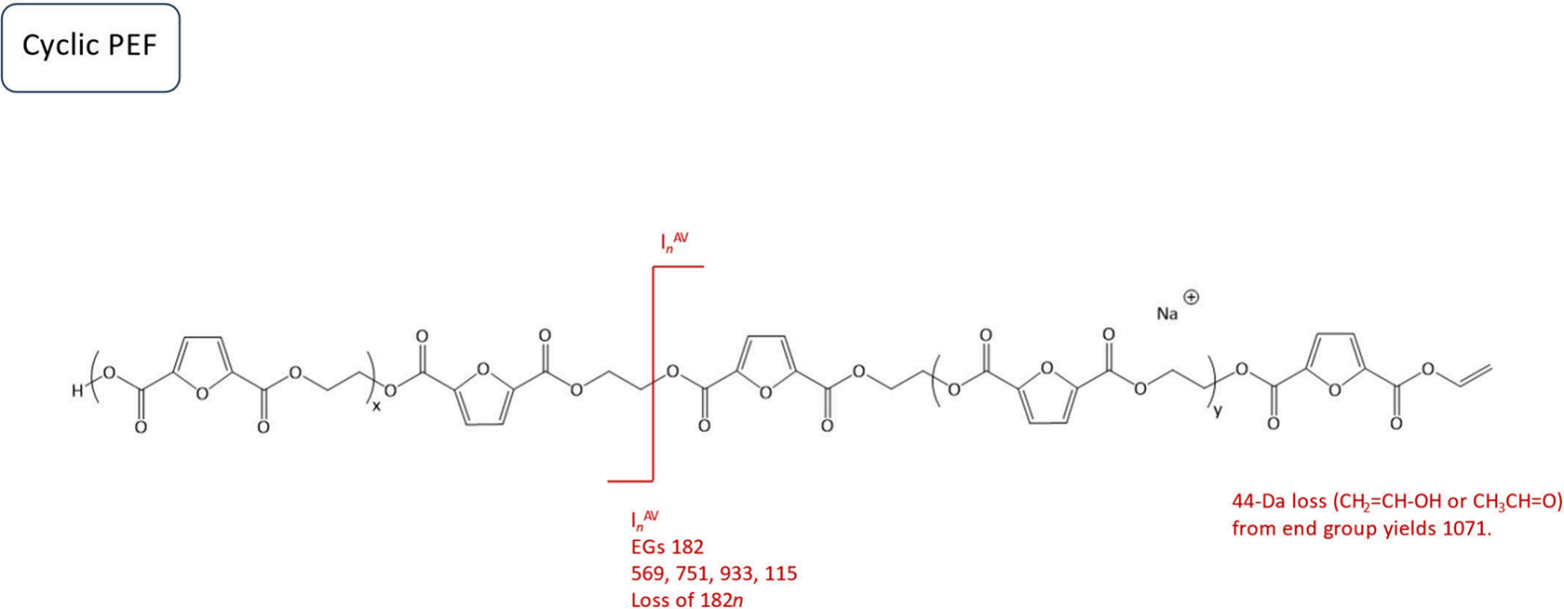
[M + Na]^+^ from Cyclic PEF Polyesters and Proposed Fragmentation Mechanism after Ring Opening via 1,5-H Rearrangement The *m/z* values of the main fragments are displayed in the scheme. *l*_n_, linear fragments; AV, vinyl acid.

The MALDI-MS/MS spectrum of the cyclic PEF polyester has been displayed in Figure 6. The MS/MS spectrum of cyclic PEF shows the repeating units of PEF and a mass loss of EG_2_. The loss of EG_2_ from the polymer chain (CH_2_=CH–OH or CH_3_CH=O) yields *m*/*z* 1071. Cyclic architectures necessitate the cleavage of at least two bonds for fragment production, in contrast to linear structures. The cyclic PEF polyester must fragment via a ring opening mechanism, leading to a linear isomer with an unsaturated chain end, as illustrated in Scheme 3. The cyclic PEF polymeric chain was cleaved into two linear fragments, both bearing vinyl and acid end-groups.

**Figure 7:**
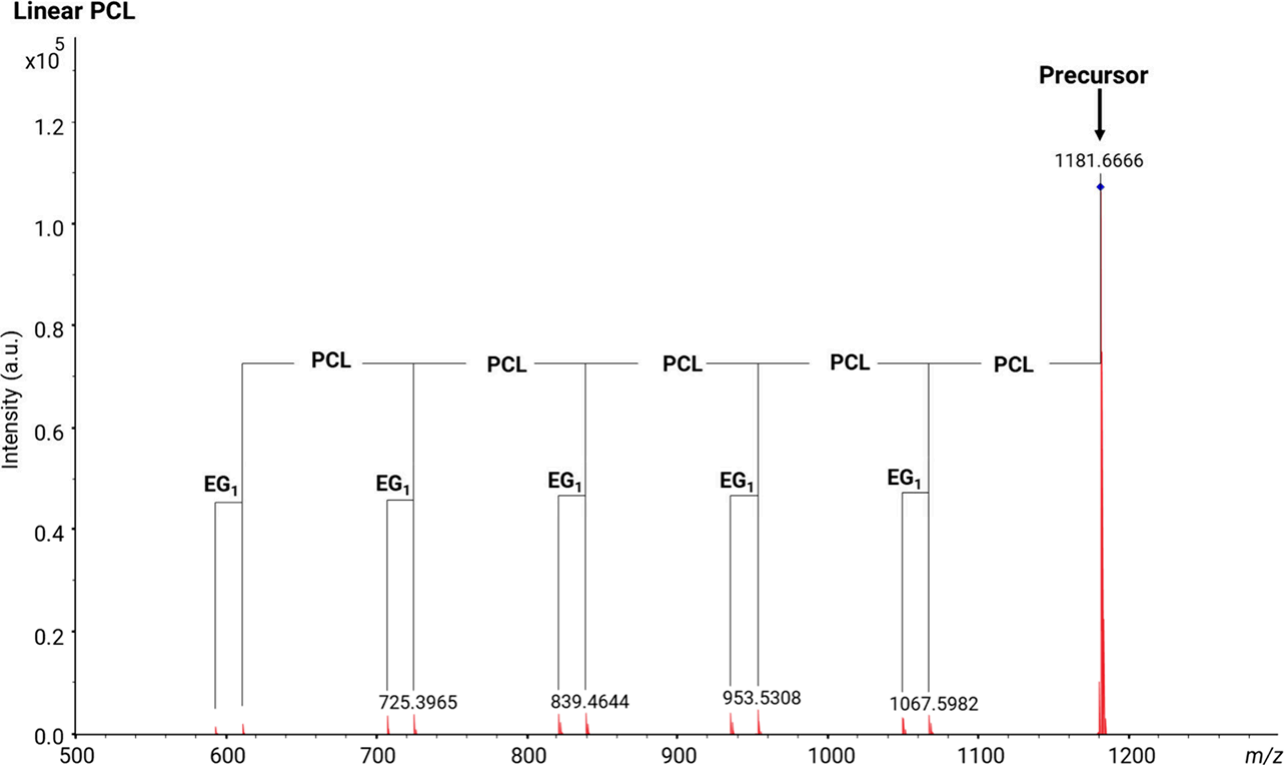
MALDI-MS/MS spectrum of [M + Na]^+^ from linear PCL polyester (*m*/*z* 1182) showing fragment series from the loss of EG_1_ and *n* repeating units.

**Scheme 4:**
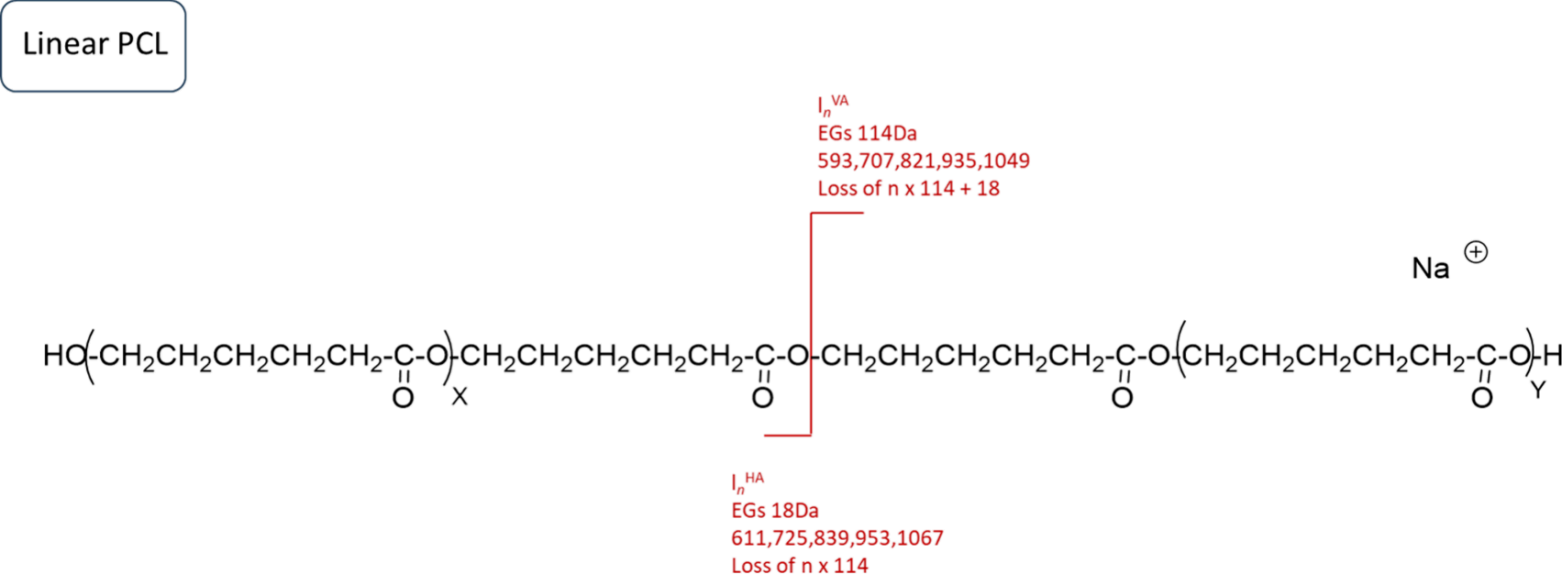
[M + Na]^+^ from Linear PCL Polyester and Proposed Fragmentation Mechanism The *m/z* values of the main fragments are displayed in the scheme. *l*_n_, linear fragments; AV, vinyl acid; HA, acid hydroxyl.

Figure 7 displays the MALDI-MS/MS spectrum of the linear neat PCL polymer. The precursor ion with an *m*/*z* value of 1182 was selected for tandem MS. The fragments observed show repeating units of PCL and loss of EG_1_. A plausible fragmentation mechanism for linear neat PCL is illustrated in Scheme 4. The linear neat PCL chain was cleaved into two linear fragments: one with vinyl acid end-groups from the terminating chain end and one with acid hydroxyl end-groups from the initiating chain end.

**Figure 8:**
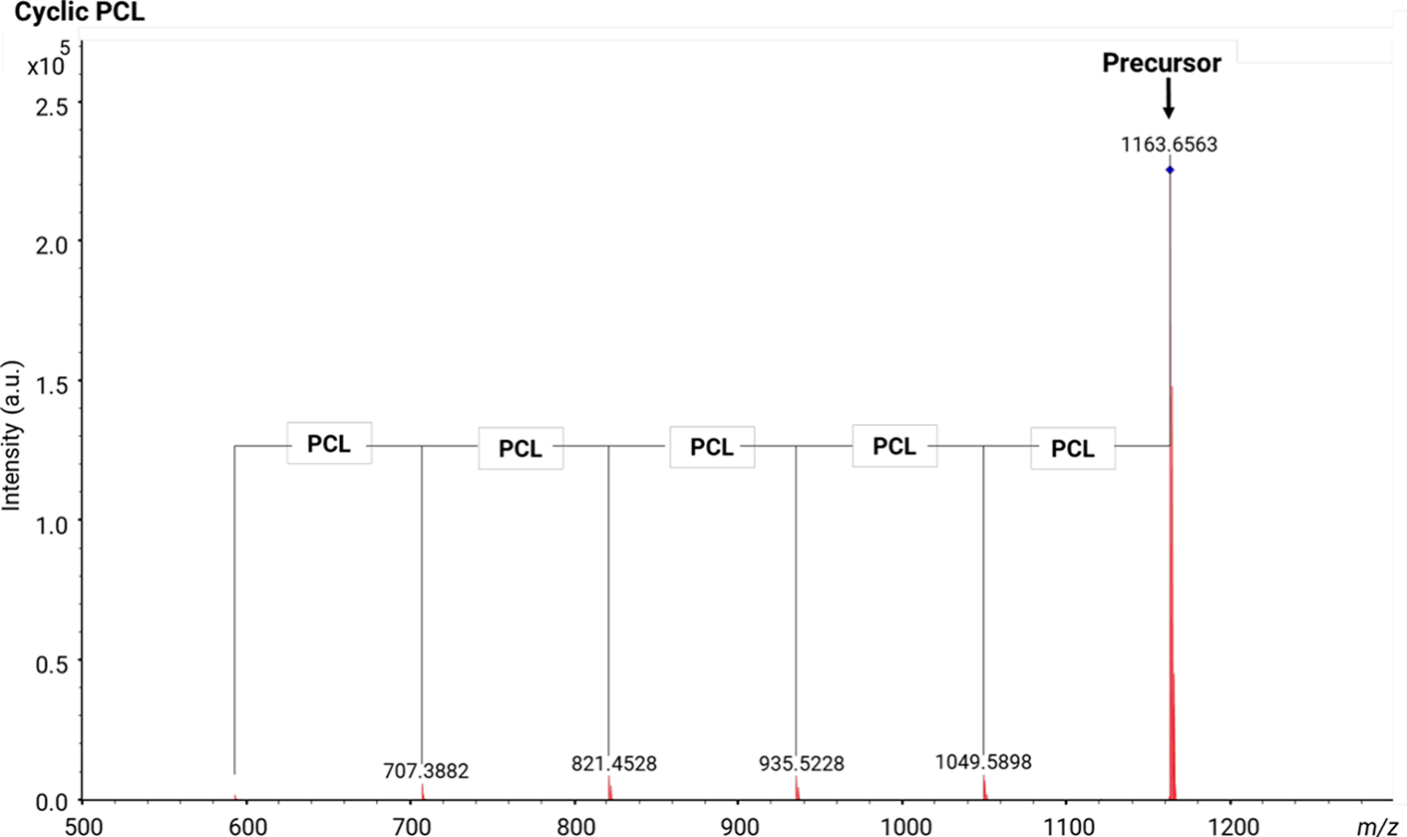
MALDI-MS/MS spectrum of [M + Na]^+^ from a cyclic PCL polyester (*m*/*z* 1163).

**Scheme 5:**
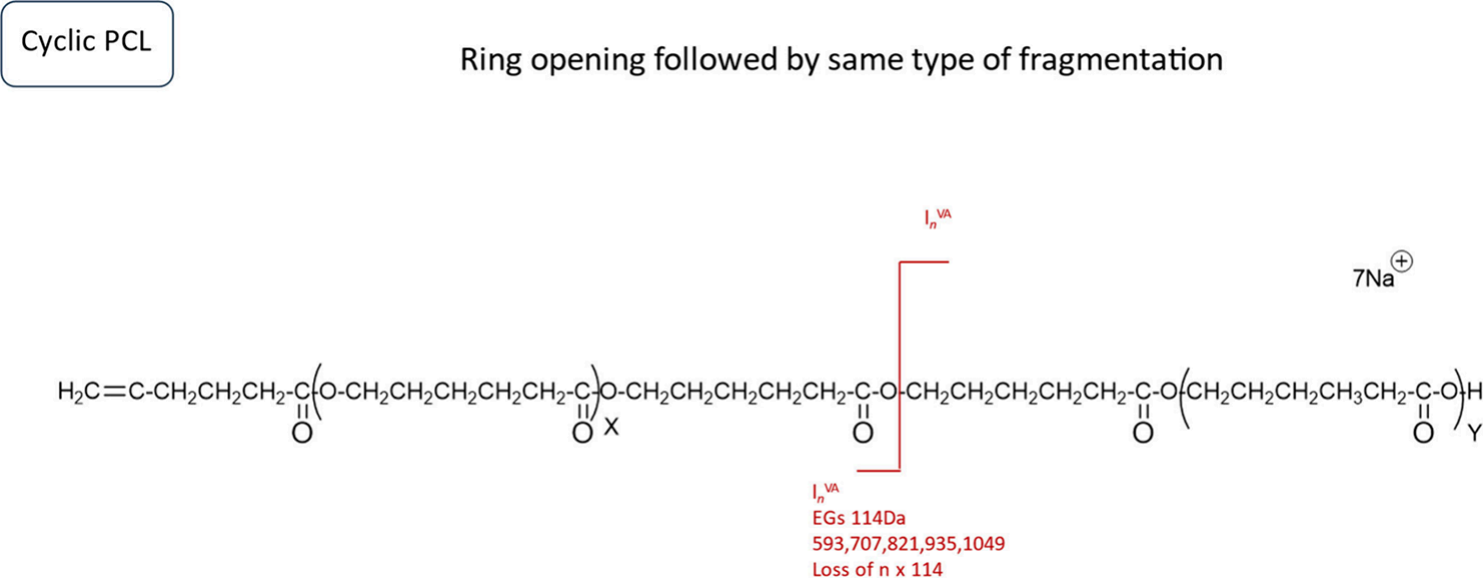
[M + Na]^+^ from Cyclic PCL Polyester and Proposed Fragmentation Mechanism The *m/z* values of the main fragments are displayed in the scheme. *l*_n_, linear fragments; AV, vinyl acid.

The MALDI-MS/MS spectrum of the cyclic neat PCL shows just the repeating units of PCL, as displayed in Figure 8. The fragments observed can be explained via ring opening followed by chain scission to two linear fragments with vinyl acid end-groups attached, as described in Scheme 5; both of these steps can proceed via 1,5-H rearrangement.

**Scheme 6:**
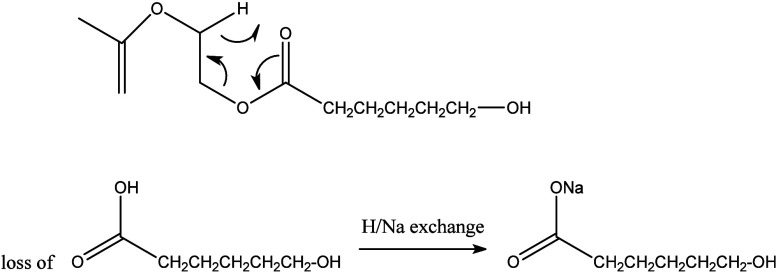
Loss of a C_6_H_11_O_3_Na (154 Da) Unit during Fragmentation of [M + Na]^+^ from the P5050 Block Copolymer Note that H/Na exchange can occur before or after fragmentation. The remaining fragment ion is protonated.

**Scheme 7:**
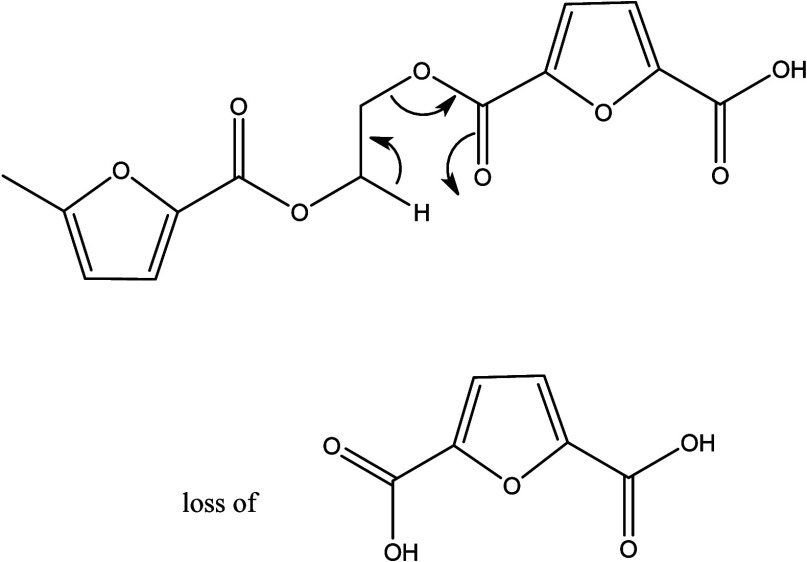
Loss of a C_6_H_4_O_5_ (156 Da) Unit during Fragmentation of [M + Na]^+^ from the P5050 Block Copolymer

**Figure 9:**
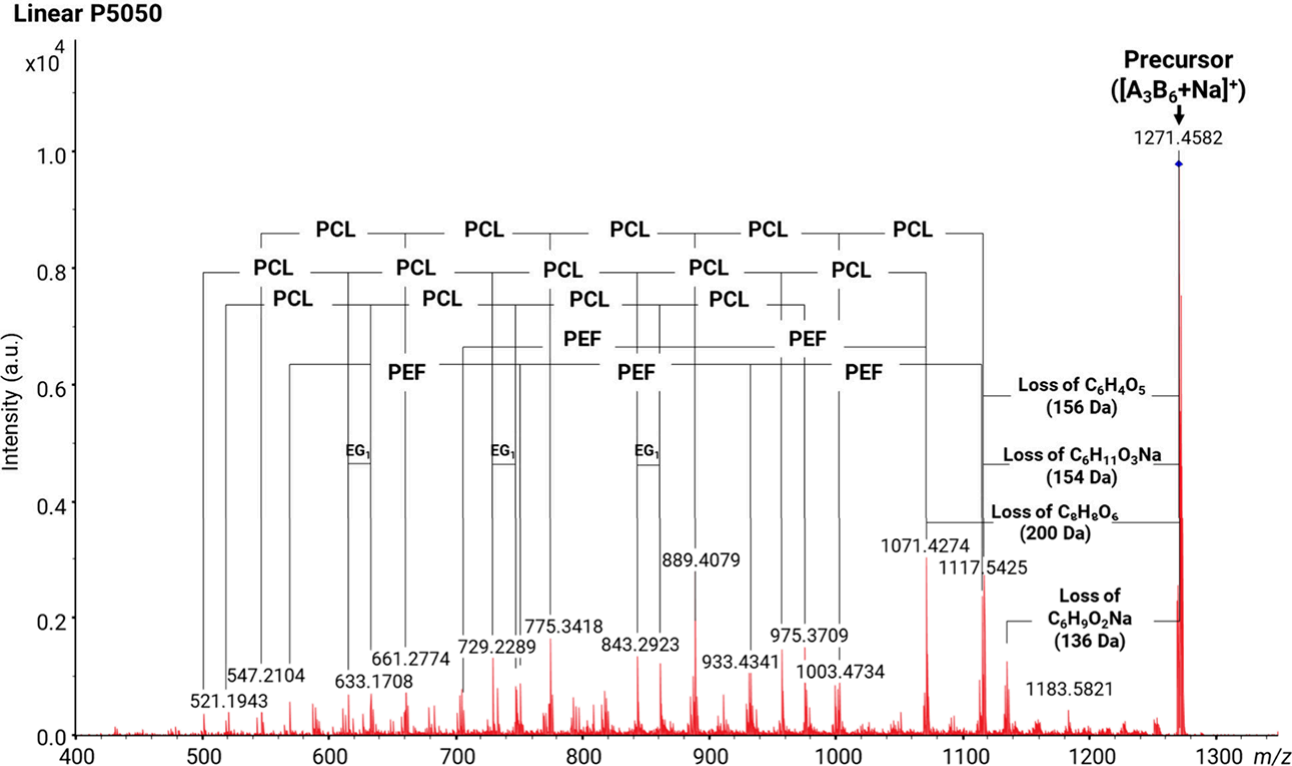
MALDI-MS/MS spectrum of [M + Na]^+^ of the linear P5050 block copolymer (*m*/*z* 1271; [A_3_B_6_ + Na]^+^).

Among the block copolymers, MALDI-MS/MS spectra of the P5050 copolymer have been selected for discussion and the remaining MALDI-MS/MS spectra of the other block copolymers have been added to the supplementary section (Figures S1–S8). Figure 9 shows the MALDI-MS/MS spectrum of the linear P5050 block architecture for which the precursor ion with *m*/*z* = 1271 was selected. Based on its accurate *m*/*z* ratio, this ion has the composition A_3_B_6_, i.e., a longer PCL than the PEF block. Indeed, the MS/MS fragmentation confirms that more repeating units of PCL were attached to PEF units as a block structures. As explained in Section 3.1, the increase in the ε-CL content increases the side chain length of the block copolymers. Losses of C_6_H_11_O_3_Na (154 Da, sodiated ε-hydroxy hexanoic acid, Scheme 6) and C_6_H_4_O_5_ (156, 2,5-FDCA, Scheme 7) and of a complete PEF monomer (200 Da, 2,5-FDCA + C_2_H_4_O) were observed. As annotated in Figure 9, losses composed of C_6_H_11_O_3_Na (154, characteristic of PCL) and up to five additional PCL units are observed, confirming that a substantial portion of the A_3_B_6_ precursor ion contains a continuous BBBBBB block. Similarly, losses coming from C_6_H_4_O_5_ (156, part of the PEF repeating unit) and up to three additional PEF units were observed, consistent with having a chain segment with an AAA block.

**Figure 10:**
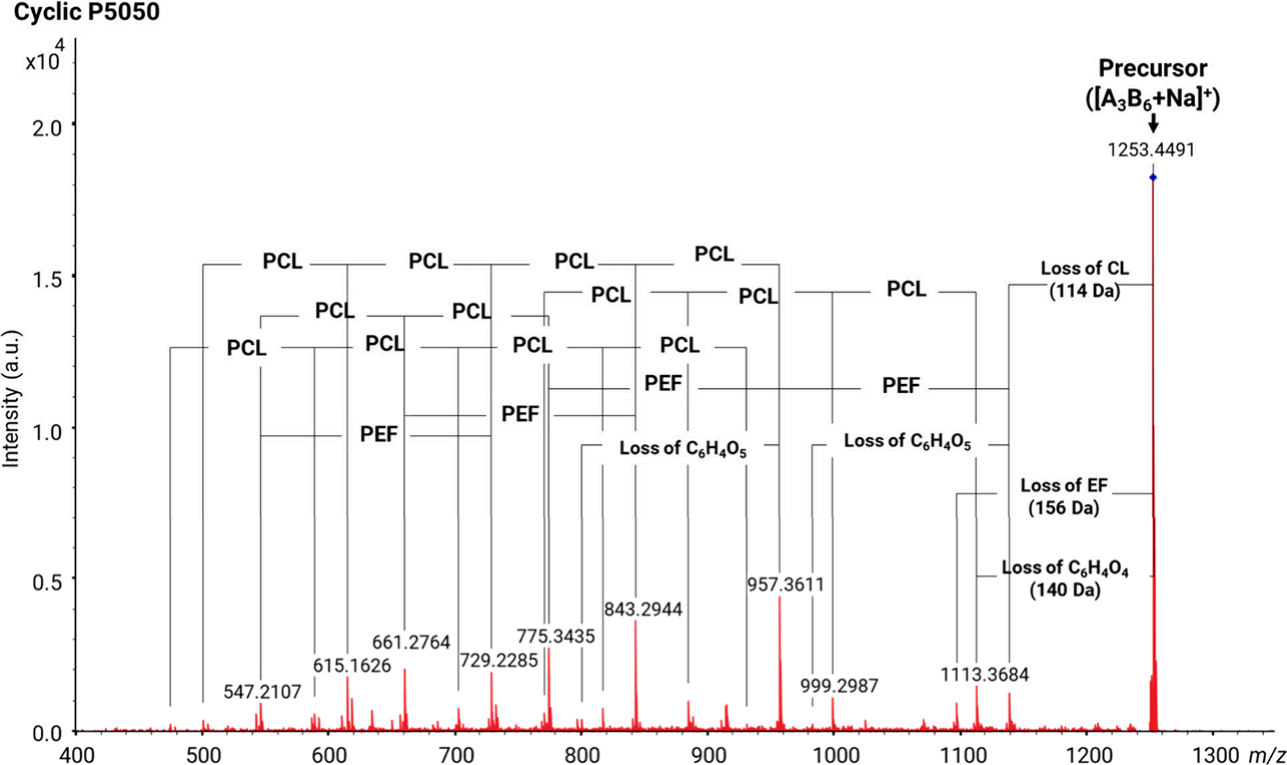
MALDI-MS/MS spectrum of [M + Na]^+^ of the cyclic P5050 block copolymer (*m*/*z* 1253; [cA_3_B_6_ + Na]^+^). The C_6_H_4_O_4_ moiety (140 Da) most likely is 2,5-furan monocarboxylic acid monoaldehyde. Such an end-group can be formed upon the ring opening of the cyclic architecture.

The MALDI-MS/MS spectrum of the cyclic P5050 block copolymer is displayed in Figure 10. Like the linear P5050 block copolymer, the spectrum shows a similar sequence of repeating units of PEF and PCL attached to both ends.

### 3.3 NMR Analysis

**Figure 11:**
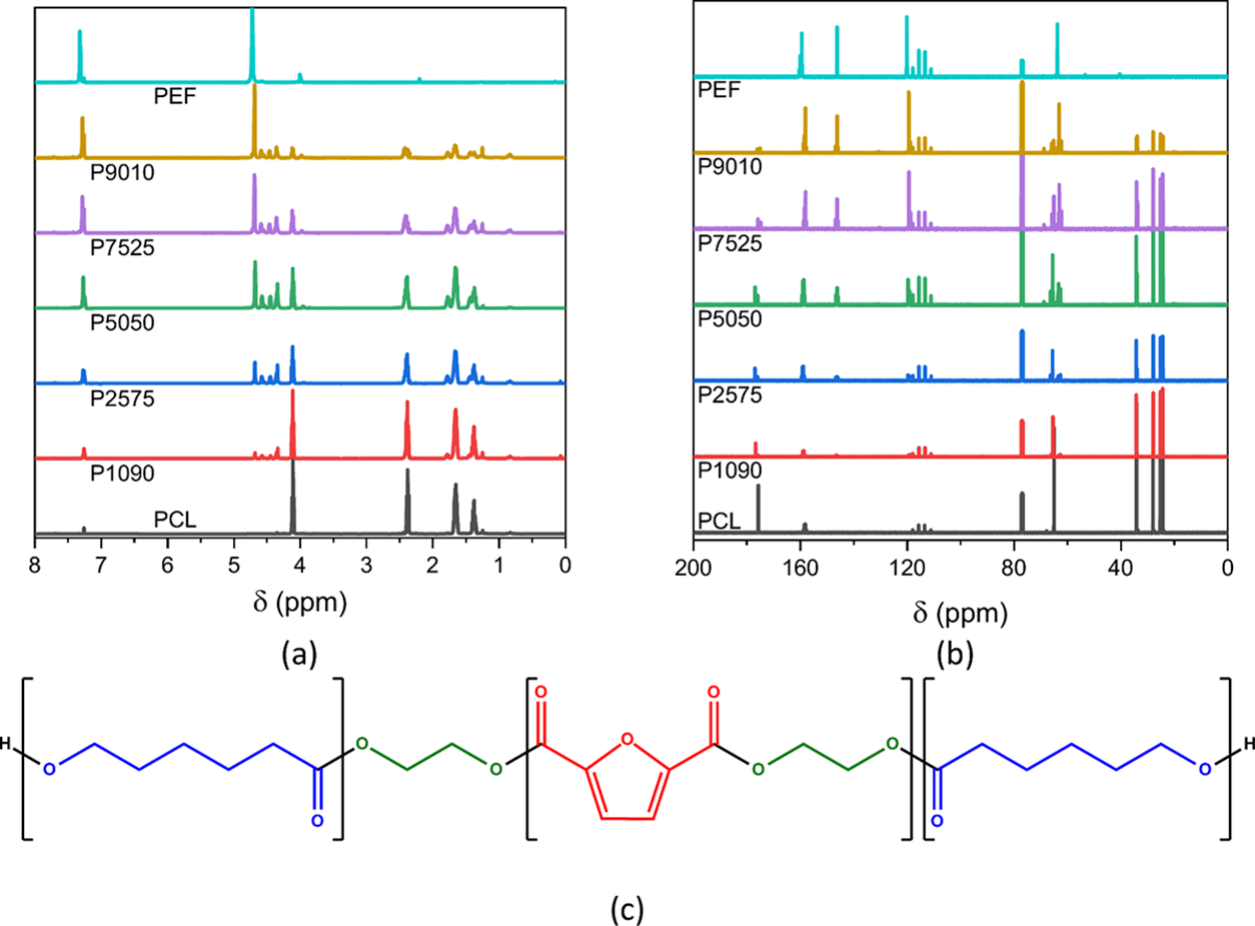
(a) ^1^H and (b) ^13^C spectra of all synthesized PEF–PCL polymers. In the ^13^C spectra, the signals at approximately 77, 111–117, and 160 ppm are attributed to the residual signals of CDCl_3_ and TFA-*d*_1_.

In the NMR spectra of the PEF–PCL block copolymers (Figure 11), expected resonance signals corresponding to PEF and PCL segments can be observed. More specifically, the following signals are observed in the ^1^H NMR spectra for PCL: 4.11 ppm corresponding to the −OCH_2_– protons, 2.38 ppm corresponding to the −C(O)CH_2_– protons, and 1.65 and 1.37 ppm corresponding to the −CH_2_CH_2_CH_2_– protons. For PEF, the following signals are observed: 4.72 and 7.35 ppm for −OCH_2_CH_2_O– and CH aromatic protons of the furan ring, respectively. In the ^13^C spectra, the following peaks are observed for PCL: 175.7 ppm attributed to the C(O) ester carbon atom, 65.0 ppm attributed to the −OCH_2_– carbon atom, 34.2 ppm attributed to the −C(O)CH_2_– carbon atom, and 28.0, 25.3, and 24.4 ppm attributed to the −CH_2_CH_2_CH_2_– carbon atoms. For PEF, the following peaks are observed: 159.5 ppm attributed to the C(O) ester carbon atom, 146.3 and 120.2 ppm attributed to the carbon atoms of the furan ring, and 63.8 ppm attributed to the −OCH_2_CH_2_O– carbon atoms. The composition of the copolymers was calculated by integrating the peaks attributed to the −OCH_2_CH_2_O– groups of ethylene glycol from PEF segments and the −OCH_2_– methylene group of PCL segments. The copolymers present slightly higher PCL content than the feed ratio (Table 4).

**Table 4:** Calculated PEF–PCL Composition and Microstructure

	composition (%)		microstructure		
copolymer	PEF segments	PCL segments	*B*	*L*_EF_	*L*_CL_
P9010	85	15	0.29	65.3	3.6
P7525	70	30	0.22	32.0	5.3
P5050	50	50	0.28	10.8	5.4
P2575	22	78	0.39	4.3	6.4
P1090	6	94	0.49	2.5	11.2

**Figure 12:**
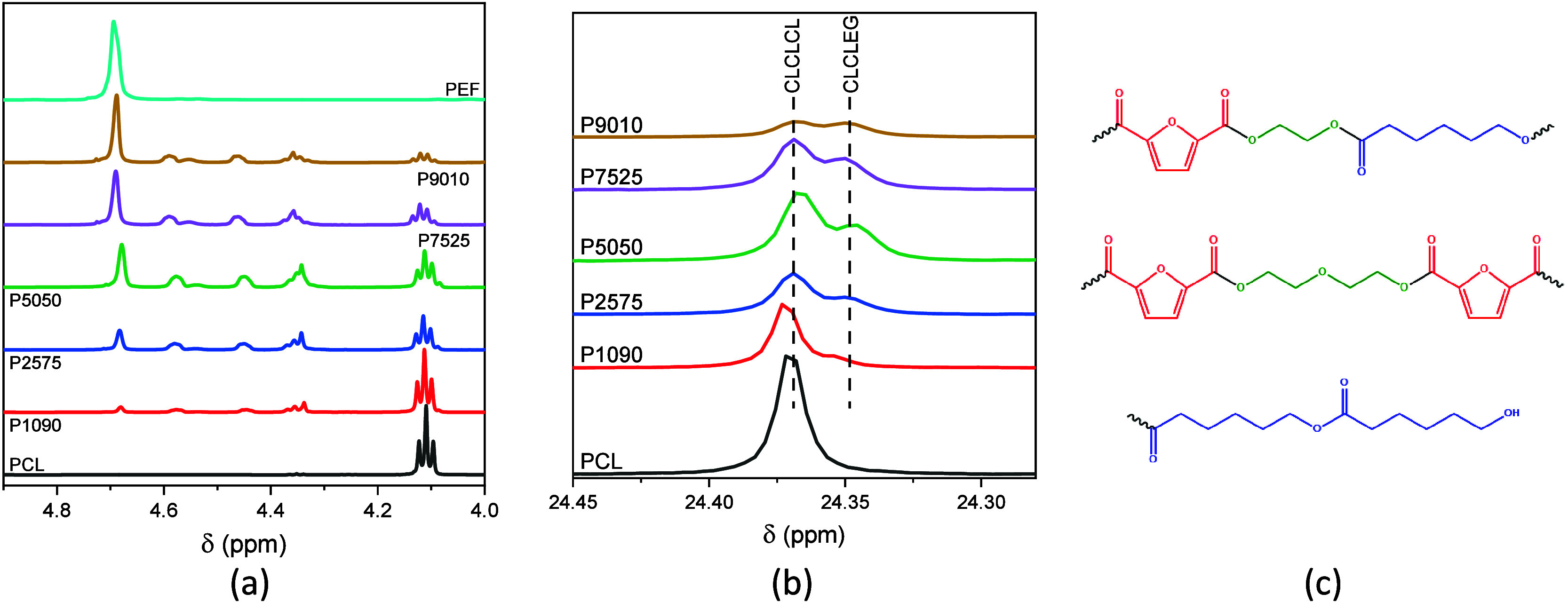
Zoomed-in regions of (a) the ^1^H and (b) the ^13^C NMR spectra of the copolymers. (c) Possible microstructures in the copolymers.

According to the literature,^27^ the peaks in Figure 12a are attributed to units of ethylene glycol having a caprolactone unit on one side and a furan ring on the other side, diethylene glycol units, and −CH_2_OH end chain groups (Figure 12c). The presence of the former signals indicates the successful copolymerization between PEF and ε-CL. According to Wang et al.,^27^ furan dicarboxylate–caprolactone and caprolactone–ethylene glycol–caprolactone sequences also give rise to peaks in this region. The microstructure was deduced by the degree of randomness *B*, which is used when symmetric and nonsymmetric monomers are copolymerized. *B* is calculated from the signal around 24 ppm of the PCL segments (Figure 12b) by comparing the integration of the peak arising from PCL segments (CLCLCL) to the integration of all CL patterns (CLCLCL and CLCLEG, i.e., ε-CL units bonded to EG), as described in the experimental part.^39^ The degree of randomness ranges from 0.2 to 0.5, indicating blocky structures (*B* < 1) with an average PEF sequence length increasing with increasing PEF content (Table 4).

## 4 Conclusion

In this study, we performed the synthesis of biobased PEF–PCL block copolymers with various mass ratios using ROP of ε-CL in the presence of PEF. Our results demonstrate that increasing the ε-CL content produced block copolymers with increasing molecular weight. MALDI-MS measurements in the mass range *m*/*z* 1000–1500 confirmed the successful formation of the block copolymers with PEF/PCL mass ratios of 10/90, 25/75, 50/50, 75/25, and 90/10 w/w. Linear and cyclic block copolymers were further characterized using MALDI-MS/MS. The fragmentation patterns detected with MALDI-MS/MS indicated a small block sequence of the synthesized block copolymers. NMR analysis confirms the composition and blocky structures for the synthesized PEF–PCL block copolymers. The incorporation of ε-CL into the copolymers is expected to reduce the ductility and enhance the flexibility of neat PEF due to an increase in chain mobility. These copolymers are anticipated to exhibit improved heat resistance and thermal stability compared to neat PCL. The copolymers synthesized in this study are found to be promising sustainable alternatives to fossil-based plastics, with tailored thermal and mechanical properties for food packaging and industrial applications, which require durability and adaptability.

## Supporting Information Available

Tables featuring the theoretical *m*/*z* values for PEF and PCL polymers, a table detailing the most abundant end-groups of PEF and PCL and their theoretical *m*/*z* values, and MS/MS spectra for block copolymers P1090, P2575, P7525, and P9010 in both linear and cyclic form (PDF)
