# Supplementary material for: Synthesis and Characterization of Poly(ethylene furanoate)/Poly(ε-caprolactone) Block Copolymers
Source: J Am Soc Mass Spectrom. 2025 Jan 9;36(2):286–98. doi: 10.1021/jasms.4c00397 (PMC11809208; doi:10.1021/jasms.4c00397)
Supplement: Supplementary file 1 — js4c00397_si_001.pdf [file js4c00397_si_001.pdf]

Supporting Information

Synthesis and Characterization of Poly(ethylene furanoate)/poly(ε-caprolactone) block co-polymers

Johan Stanley[1‡], Lidia Molina-Millán[2‡], Chrys Wesdemiotis[3], Ron M.A. Heeren[2*], Alexandra Zamboulis[1], Lidija Fras Zemljič [4], Dimitra A. Lambropoulou [5,6] and Dimitrios N, Bikiaris[1]

[1] Laboratory of Chemistry and Technology of Polymers and Colors, Department of Chemistry, Aristotle University of Thessaloniki, GR-54124 Thessaloniki, Greece, johansta@chem.auth.gr (J.S.), azampouli@chem.auth.gr (A.Z), dbic@chem.auth.gr (D.N.B.).

[2] The Maastricht MultiModal Molecular Imaging Institute (M4i), Division of Imaging Mass Spectrometry, Maastricht University, Universiteitssingel 50, 6229 ER Maastricht, The Netherlands, l.molinamillan@maastrichtuniversity.nl (L.M.M.), r.heeren@maastrichtuniversity.nl (R.M.A.H.)

[3] Department of Polymer Science and Department of Chemistry, The University of Akron, Akron, Ohio 44325, United States, wesdemi@uakron.edu (C.W.)

[4] Faculty of Mechanical Engineering, University of Maribor, SI-2000 Maribor, Slovenia. lidija.fras@um.si (L.F.Z).

[5] Laboratory of Environmental Pollution Control, Department of Chemistry, Aristotle University of Thessaloniki, GR–541 24 Thessaloniki, Greece; dlambro@chem.auth.gr (D.L.B).

[6] Center for Interdisciplinary Research and Innovation (CIRI-AUTH), Balkan Center, GR-570 01 Thessaloniki, Greece.

‡ These authors have contributed equally

* Corresponding Author

Table S1. Theoretical *m/z* values for the $[M+Na]^+$ ions of PEF polymer (cyclic and linear forms). The monoisotopic values were considered for the calculation of the theoretical *m/z*.

	PEF		
**Degree of polymerization (n)**	**$[M+Na]^+$ (linear, with EG_1)**	**$[M+Na]^+$ (linear, with EG_3)**	**$[M+Na]^+$ (cyclic)**
2	405.0428	449.0691	387.0323
3	587.0644	631.0906	569.0538
4	769.0859	813.1121	751.0753
5	951.1074	995.1336	933.0968
6	1133.1289	1177.1552	1115.1184
7	1315.1505	1359.1767	1297.1399
8	1497.1720	1541.1982	1479.1614
9	1679.1935	1723.2197	1661.1829
10	1861.2150	1905.2413	1843.2045
11	2043.2366	2087.2628	2025.2260
12	2225.2581	2269.2843	2207.2475

Table S2. Theoretical *m/z* values for the $[M+Na]^+$ ions of PCL polymer (cyclic and linear forms). The monoisotopic values were considered for the calculation of the theoretical *m/z*.

	PCL	
**Degree of polymerization (n)**	**$[M+Na]^+$ (linear, with EG_1)**	**$[M+Na]^+$ (cyclic)**
2	269.1359	251.1254
3	383.2040	365.1935
4	497.2721	479.2615
5	611.3402	593.3296
6	725.4083	707.3977
7	839.4763	821.4658
8	953.5444	935.5339
9	1067.6125	1049.6019
10	1181.6806	1163.6700
11	1295.7487	1277.7381
12	1409.8167	1391.8062
13	1523.8848	1505.8743
14	1637.9529	1619.9423
15	1752.0210	1734.0104
16	1866.0891	1848.0785
17	1980.1571	1962.1466
18	2094.2252	2076.2147

Table S3. Theoretical *m/z* values for the monomeric units of PEF and PCL and the main end-groups for these polymers. The monoisotopic values were considered for the calculation of the theoretical *m/z*.

Name	Formula	Theoretical *m/z*
**PEF monomer**	$C_8H_6O_5$	182.0215
**PCL monomer**	$C_6H_{10}O_2$	114.0681
**EG_1**	H_2O	18.0106
**EG_2**	C_2H_4O	44.0262
**EG_3**	$C_2H_6O_2$	62.0368
**EG_4**	$C_{10}H_8O_5$	208.0372
**EG_5**	$C_4H_{10}O_3$	106.0630

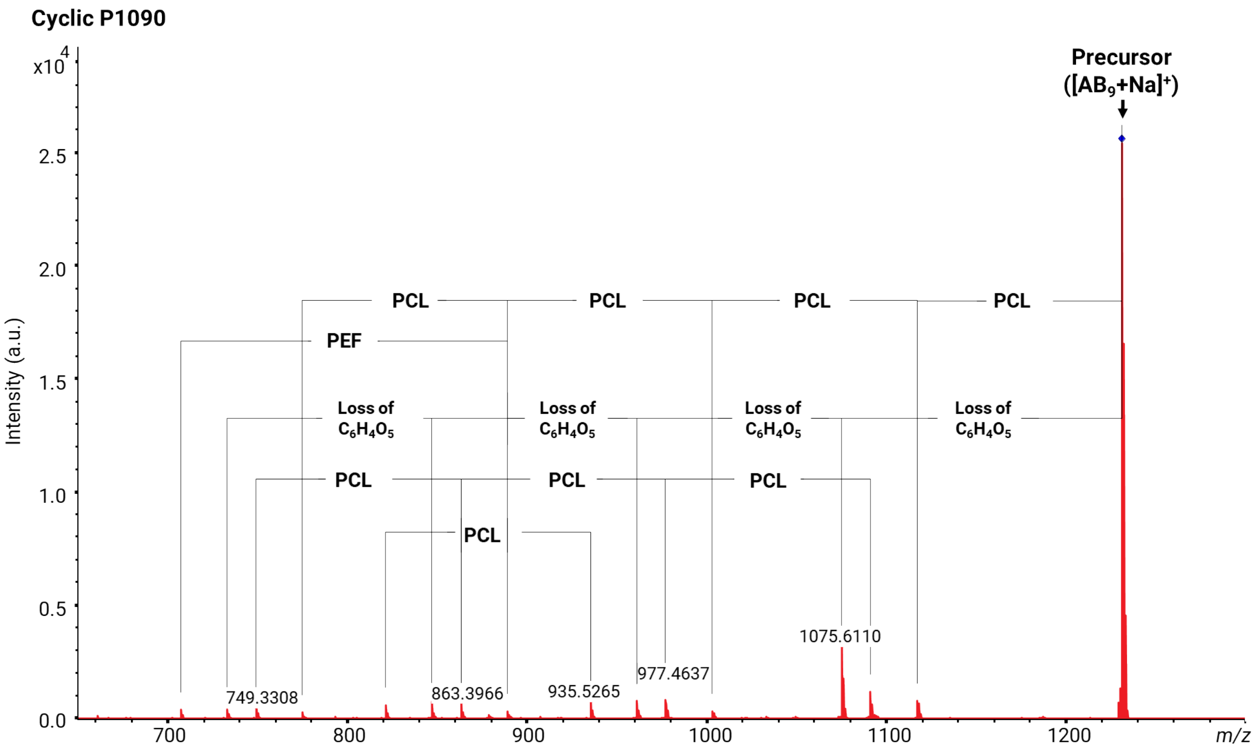

Figure S1. MALDI-MS/MS spectrum of [M + Na]$^+$ of the cyclic P1090 block co-polymer (*m/z* 1231; $[AB_9+Na]^+$).

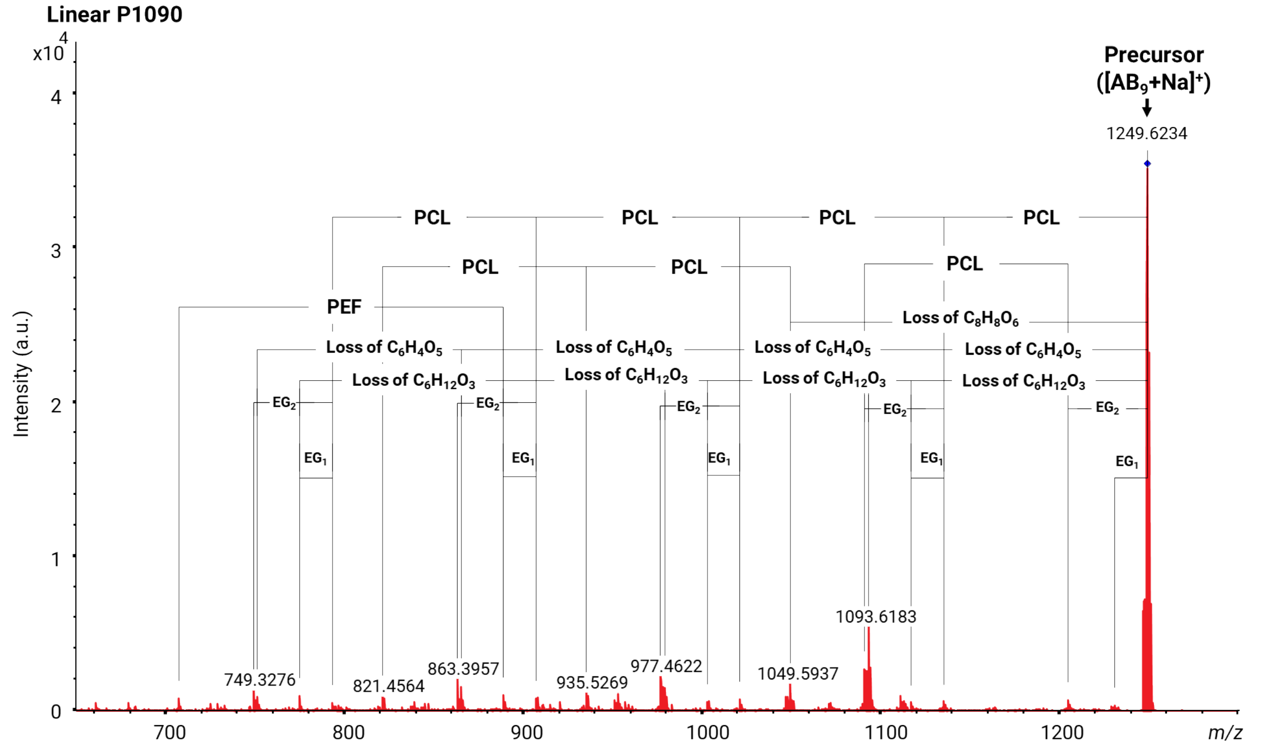

Figure S2. MALDI-MS/MS spectrum of [M + Na] $^+$ of the linear P1090 block co-polymer (*m/z* 1249; $[AB_9+Na]^+$).

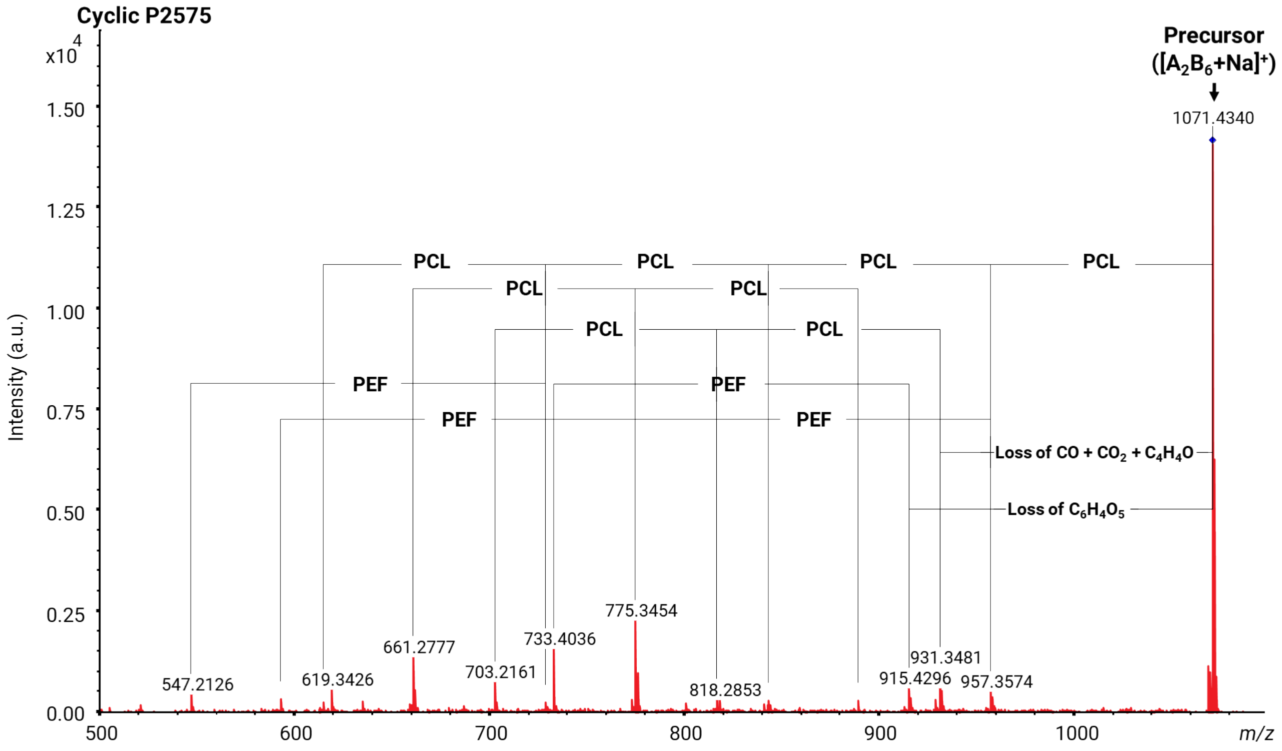

Figure S3. MALDI-MS/MS spectrum of [M + Na] $^+$ of the cyclic P2575 block co-polymer (*m/z* 1071; $[A_2B_6+Na]^+$).

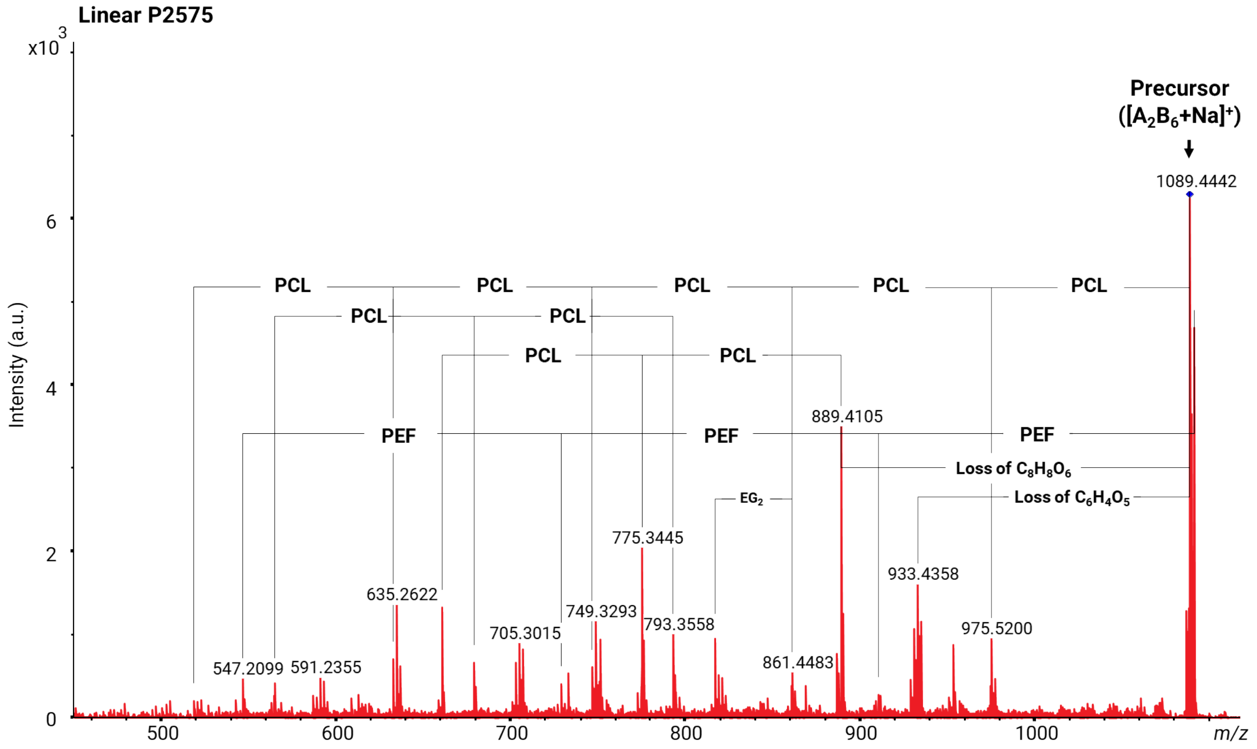

Figure S4. MALDI-MS/MS spectrum of [M + Na]$^+$ of the linear P2575 block co-polymer (*m/z* 1089; $[A_2B_6+Na]^+$).

Figure S5. MALDI-MS/MS spectrum of [M + Na]$^+$ of the cyclic P7525 block co-polymer (*m/z* 1275; $[A_5B_3+Na]^+$).

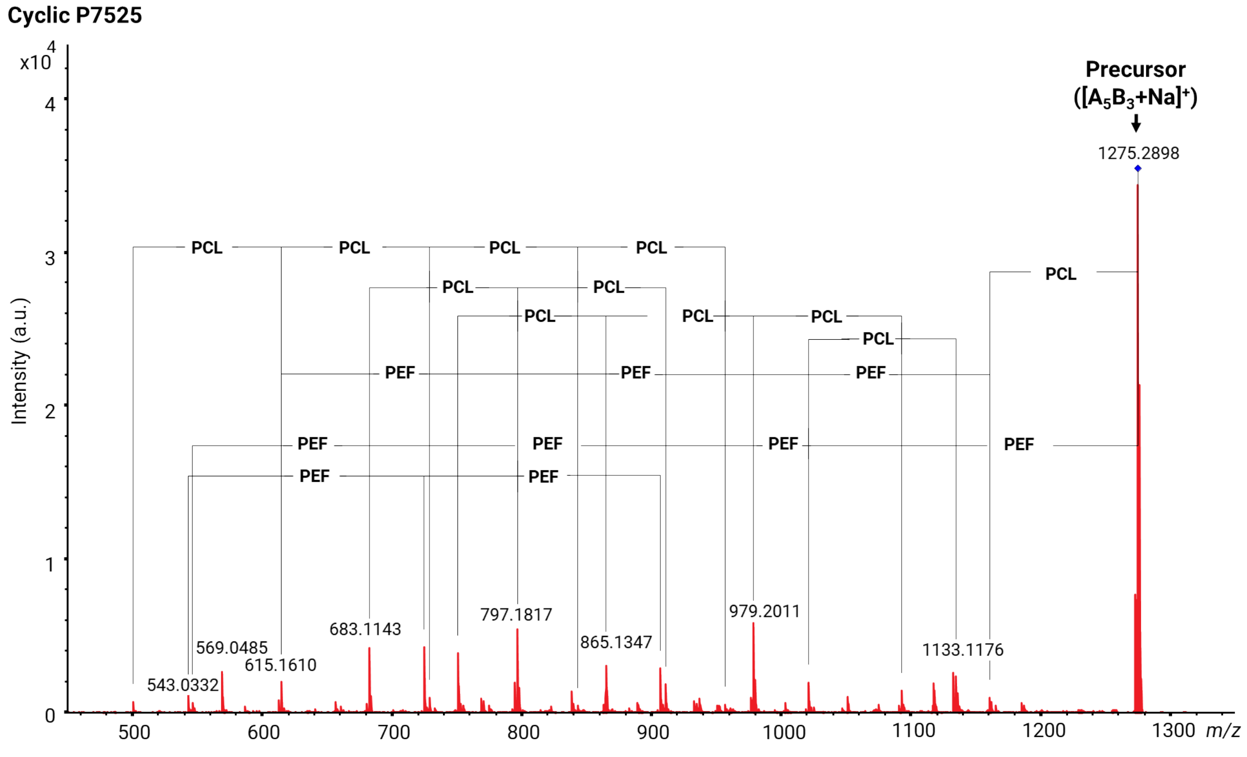

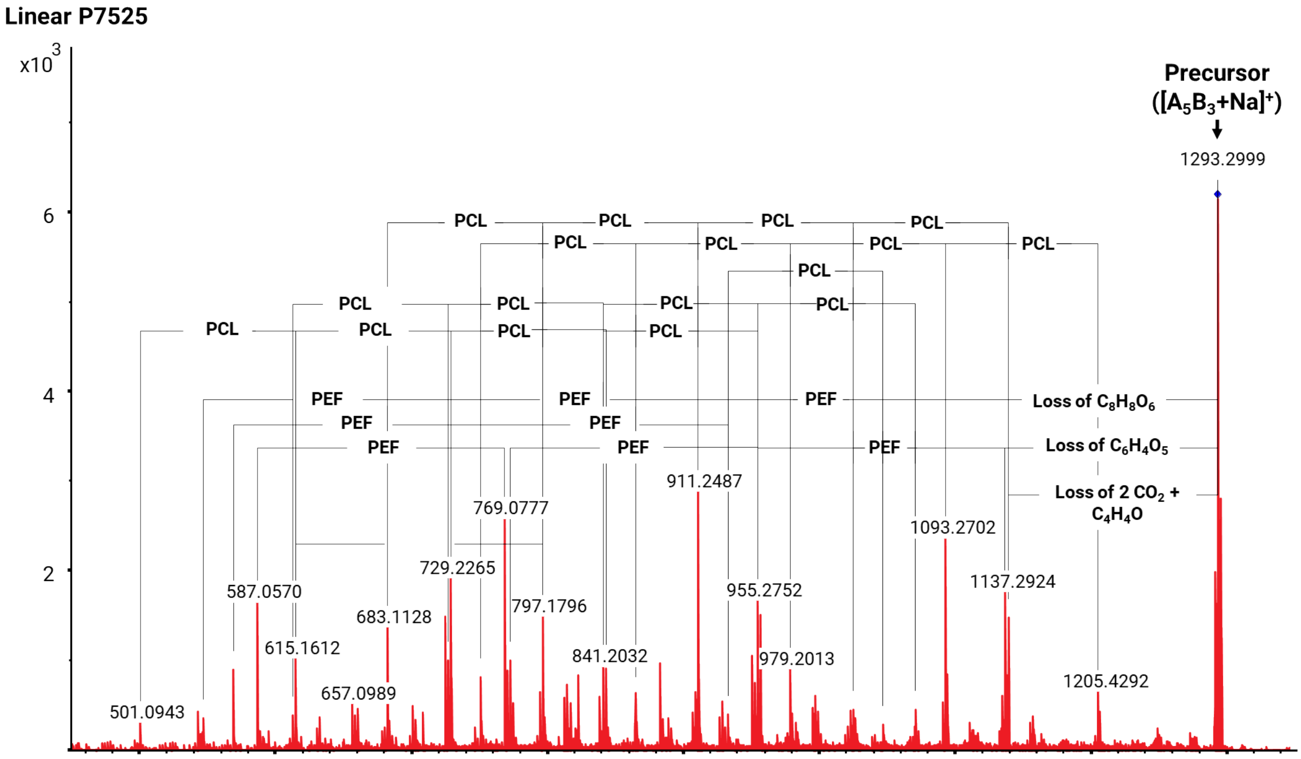

Figure S6. MALDI-MS/MS spectrum of [M + Na] $^+$ of the linear P7525 block co-polymer (*m/z* 1293; $[A_5B_3+Na]^+$).

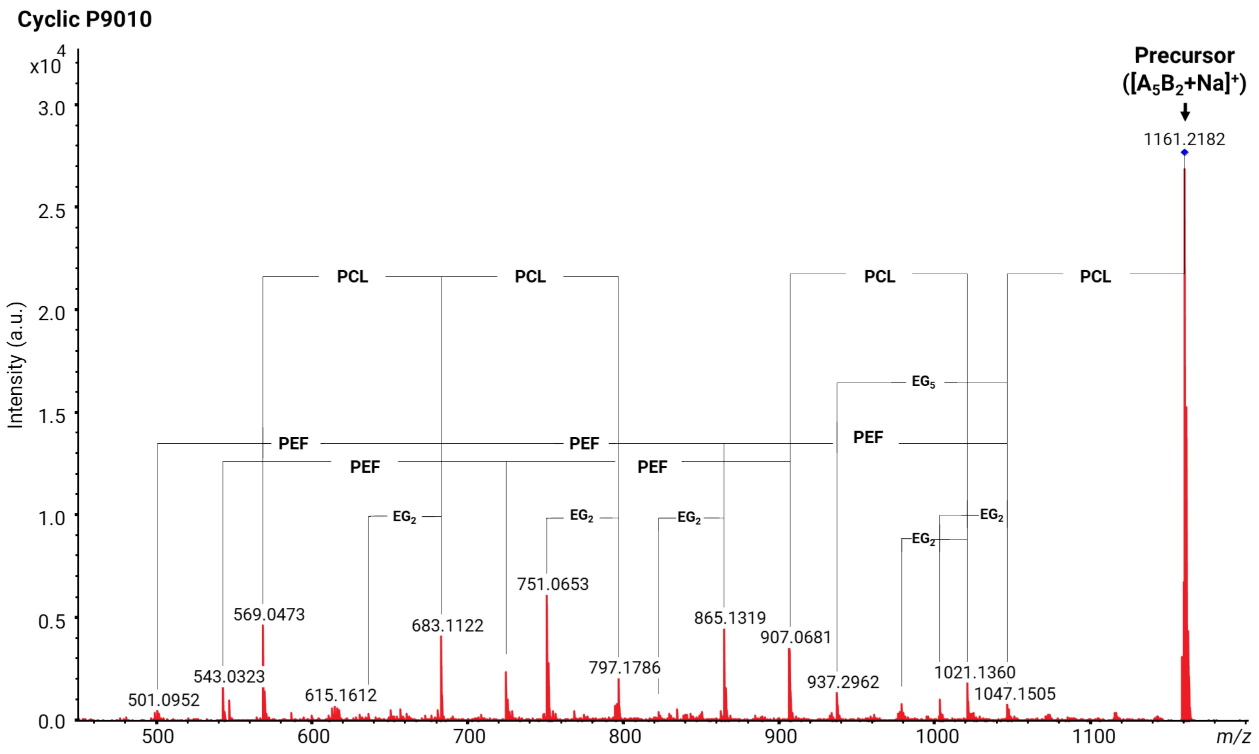

Figure S7. MALDI-MS/MS spectrum of [M + Na] $^+$ of the cyclic P9010 block co-polymer (*m/z* 1161; $[A_5B_2+Na]^+$).

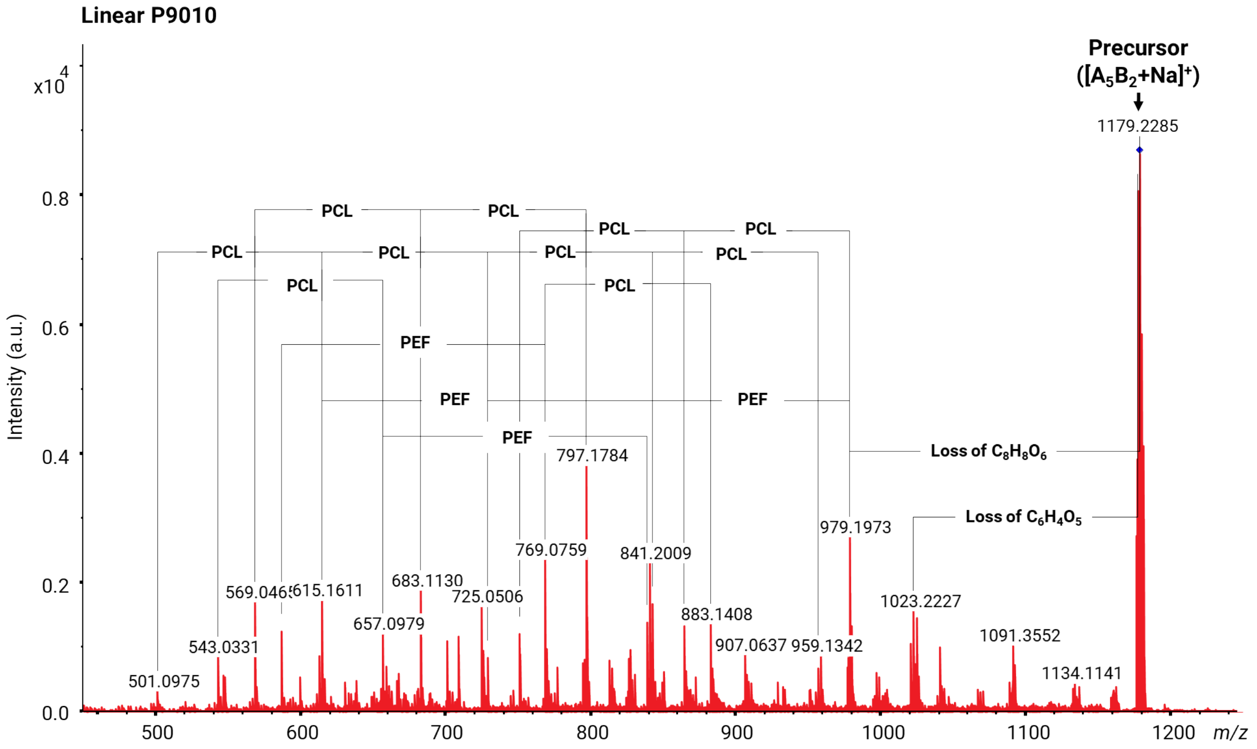

Figure S8. MALDI-MS/MS spectrum of [M + Na] $^+$ of the linear P9010 block co-polymer (*m/z* 1179; $[A_5B_2+Na]^+$).
